# Cytogenetic Studies in Malignant Lymphomas: A Study of 28 Cases

**DOI:** 10.1038/bjc.1971.93

**Published:** 1971-12

**Authors:** V. Coutinho, C. Bottura, R. P. Falcao

## Abstract

**Images:**


### CYTOGENETIC STUDIES IN MALIGNANT LYMPHOMAS: A STUDY OF 28 CASES

V. COUTINHO, C. BOTTURA AND R. P. FALCAO

*From the Department of Clinical Medicine, Faculty of Medicine, Ribeirao Preto, São Paulo, Brazil*

Received for publication September 13, 1971

**SUMMARY.—Chromosome studies were carried out, by a direct method, in 28 subjects with malignant lymphomas. Lymph node cells were analysed in 24, ascitic fluid sediments in 3 and bone marrow cells in 1. Chromosome abnormalities, both numerical and structural, were found in 12 of 14 cases of well differentiated and poorly differentiated lymphocytic lymphomas, and reticulum cell sarcomas, and in 8 of 14 cases with Hodgkin's disease. The karyotypes were different from case to case and there was no correlation with the histology. In individual cases the abnormalities followed a clonal pattern indicating a common precursor for the abnormal cells. The modal number of chromosomes was near-diploid in the lymphocytic lymphomas and reticulum cell sarcomas.**

**Hodgkin's disease showed two main features; a predominant population of cells with normal karyotype accompanied by a small number of cells of an abnormal clone with a predominantly hypertriploid number of chromosomes. It is suggested that the first population represents mitoses in the surrounding lymphocytes. Its cytogenetic normality is an argument against its neoplastic origin, and they could be components of an inflammatory reaction, the true neoplastic cells being the abnormal reticulum cells.**

ALTHOUGH chromosome abnormalities have previously been reported in malignant lymphomas (Tjio *et al.*, 1963; Sandberg *et al.*, 1964; Sasaki *et al.*, 1965; Baker and Atkin, 1965; Seif and Spriggs, 1967; Miles *et al.*, 1966; Miles, 1967; Spiers and Baikie, 1968; Millard, 1968), the available data is scarce in comparison with the large number of cytogenetic studies in leukaemia and the similar incidence of both diseases. Approximately 80% of cases showed chromosome abnormalities which were represented by abnormal clones, with a modal number of chromosomes in the diploid range in lymphocytic lymphomas and reticulum cell sarcomas, and in the triploid–tetraploid range in Hodgkin's disease. The present report describes chromosome analyses, by a direct method, in 28 patients with malignant lymphomas.

### MATERIAL AND METHODS

Chromosome preparations were carried out on lymph node cells in 24 subjects, on ascitic fluid sediments in 3 and on bone marrow cells in 1. The procedure for chromosome analysis will be briefly described. Immediately after the surgical biopsy the lymph node was divided into two halves; one half was fixed for histological sections and the other, after imprints were made, was put in a Petri dish containing 40 ml. of saline solution 0·9% NaCl containing colcemid (CIBA) 1 $\mu$g./$\mu$l., prewarmed at 37° C. Holding the block of tissue with forceps it was teased out until a suspension of cells was produced. To remove the larger fragments the material was filtered through a small mesh sieve. The resulting fine suspension was then incubated at 37° C. for 1 hour, treated with sodium citrate solution 0·95% for 20 minutes and fixed in a solution consisting of 3 vol. of absolute ethanol to 1 vol. of glacial acetic acid. Chromosome spreads were made by the air-drying technique and stained with Leishman stain diluted 1 : 10 in water. A similar procedure was employed to obtain chromosome preparations from ascitic fluid sediments and bone marrow cells. Well-spread metaphases were selected and analysed. The best ones were photographed afterwards and the karyotype studied from cutouts of enlarged prints. The karyotypes were formulated according to the Chicago Conference (1966). The nomenclature of marker chromosomes was made following the system proposed by Levan *et al.* (1964). The histological sections were classified according to Rappaport (1966), and Lukes and Butler (1966).

All patients were untreated at the time of the study, except case 17, with Hodgkin's disease.

### RESULTS

### *Lymphocytic Lymphomas and Reticulum Cell Sarcomas*

The chromosome counts are shown in Table I. A summary of the clinical symptoms, and the chromosome findings will be described below.

*Poorly differentiated lymphocytic lymphomas*

*Case* 1.—J.F., a 63-year-old man was admitted with generalized lymphadenopathy of 3 months' history. Material for chromosome analysis: lymph node;7 cells (46) normal karyotype, 3 (45, G—), 1 (45, C—).

*Case* 2.—G.C., a 42-year-old man developed generalized lymph node enlargement and splenomegaly 2 months previously. Material for chromosome analysis: lymph node; (a) normal karyotype in 12% of the cells; (b) abnormal clone hyperdiploid (47) with a large submetacentric and a small metacentric markers, monosomy # 1, trisomy # 3, extras C and E, missing F and G group chromosomes (Table II and Fig. 1).

*Case* 3.—J.T.F., a 44-year-old man was admitted to hospital with an 8 months' history of generalized lymphadenopathy and ascites. Material: lymph node; (a) normal karyotype in 80% of the cells; (b) abnormal clone hyperdiploid (49–50 chromosomes) with a large marker submetacentric, monosomy # 1, trisomy # 3, extras C, E and G (Table II and Fig. 2). Chromosome studies in 40 cells of the ascitic fluid showed normal diploid karyotype.

*Case* 4.—J.A.C., an 18-year-old man was admitted complaining of cervical and supraclavicular lymph node enlargement which had appeared 3 months before. He also had mediastinal enlargement and pleural effusion. Material: lymph node; abnormal clone pseudodiploid in all the 3 metaphases analysed (46, monosomy # 1, trisomy # 3, D—, $E_{17-18}$+). The karyotype of 60 metaphases of bone marrow aspirate was normal.

TABLE I.—*Distribution of Chromosome Counts in 14 Cases of Lymphocytic Lymphomas and Reticulum Cell Sarcomas*

| Case No. | Material | No. of metaphases counted | Chromosome number 42 | 43 | 44 | 45 | 46 | 47 | 48 | 49 | 50 | 51 | 52 | More than 52 |
|---|---|---|---|---|---|---|---|---|---|---|---|---|---|---|
| Poorly differentiated lymphocytic lymphomas | | | | | | | | | | | | | | |
| 1 | Lymph node | 30 | | | | 6 | 24 | | | | | | | |
| 2 | Lymph node | 60 | 1* | | 3* | 7* | 11*+7 | 25* | 1* | | | | | 1*(89) 1*(90) 1*(92) 1*(94) |
| 3 | Lymph node† | 40 | | | 1 | | 31 | | | 3* | 3* | 1* | 1* | |
| 4 | Lymph node | 6 | | | | | 6 | | | | | | | |
| 5 | Lymph node† | 80 | | | | 5 | 47 | 25 | 1 | | | | | 1(58) 1(72) |
| 6 | Lymph node† | 17 | | 1 | | | 9 | | | | | | | 1*(92) 2*(93) 1*(94) 1*(95) 2*(96) |
| 7 | Bone marrow | 40 | | 1* | 1* | 1*+5 | 31 | | | | | | | 1(92) |
| 8 | Ascitic fluid | 20 | | 1* | 5* | 7* | 2*+3 | | 1 | | | | | 1*(86) |
| 9 | Lymph node† | 12 | | | | | 2*+10 | | | | | | | |
| Well differentiated lymphocytic lymphomas | | | | | | | | | | | | | | |
| 10 | Lymph node† | 15 | | 1* | 2* | 3* | 6*+1 | 1* | | | | | | 1(92) |
| 11 | Lymph node | 4 | | | | 2 | 2 | | | | | | | |
| Reticulum cell sarcomas | | | | | | | | | | | | | | |
| 12 | Ascitic fluid | 100 | | | 1 | 1*+2 | 28*+21 | 1* | 2 | | 2 | 37 | 4 | 1(102) |
| 13 | Lymph node | 40 | 1 | 3 | 2 | 6 | 26 | 1 | | | | | | 1(93) |
| 14 | Ascitic fluid | 100 | 2 | 4 | 6 | 22 | 13 | 44 | 7 | 1 | | | | 1(93) |

* Represents the number of cells with marker chromosomes.

† Cases with a nodular histologic appearance. All the others were diffuse.

It is interesting that monosomy # 1 and trisomy # 3 was found in the three last cases, but the karyotypes of the abnormal stem lines were different. The large submetacentric marker found in the abnormal cells of cases 3 and 4 had similar morphology. The short arm had the length of that of the chromosome No. 1, and the long arm was approximately a third longer. In connection with the loss of one chromosome No. 1 in both karyotypes, it is possible that the marker originated from a structural rearrangement involving this chromosome.

TABLE II.—*Karyotype Analysis of Cells from Cases* 2, 3, 10 *and* 14

| | | | Chromosome distribution in groups | | | | | | | | | | |
|---|---|---|---|---|---|---|---|---|---|---|---|---|---|
| | | | A | | | B | C | D | E | | F | G | |
| Case No. | No. of cells | No. of chromosomes | 1 | 2 | 3 | 4–5 | X, 6–12 | 13–15 | 16 | 17–18 | 19–20 | 21–22, Y | mar |
| 2 | 1 | 47 | −1 | | +1 | | −1 | | +1 | +1 | −1 | −1 | sm, m |
| | 1 | 47 | −1 | | +1 | | | | | | −1 | | sm, m |
| | 1 | 47 | −1 | | +1 | | | | | +1 | −1 | −1 | sm, m |
| | 1 | 47 | −1 | | | | +1 | | | | | | sm |
| | 2 | 47 | −1 | | | | +1 | | | +1 | −1 | | sm |
| | 1 | 47 | −1 | | +1 | | +1 | | −1 | +1 | −1 | −1 | sm, m |
| 3 | 1 | 50 | −1 | | +1 | | | | | | +1 | +2 | sm |
| | 1 | 50 | −1 | | +1 | | | | ——+1—— | | | +2 | sm |
| | 1 | 50 | −1 | | +1 | | +3 | | ——+1—— | | −1 | | sm |
| 10 | 1 | 46 | | | −1 | | −1 | | | | | | st, sm |
| | 1 | 46 | | | −1 | | | | | | | | st |
| | 1 | 46 | | | | | −2 | | | | | | st, sm |
| | 1 | 46 | | | | | | | | +1 | −2 | | st |
| | 1 | 46 | +1 | | | +1 | −4 | −1 | | | | +1 | st, sm |
| | 1 | 47 | +1 | | | | −4 | | | +1 | +1 | | st, sm |
| 14 | 1 | 45 | | | −1 | | −1 | +1 | | | | | |
| | 1 | 45 | | | | −1 | | | | +1 | | −1 | |
| | 1 | 45 | | | | −1 | | +1 | | | | −1 | |
| | 1 | 45 | | | | | −1 | | −1 | | | +1 | |
| | 1 | 45 | | | | | | −1 | | | | | |
| | 1 | 45 | | | | | | | −1 | | −1 | +1 | 1 minute |
| | 1 | 45 | | | | | | | | | | −1 | |
| | 1 | 46 | | | | | | | | −1 | −1 | +2 | |
| | 1 | 46 | | | | | | | | −1 | | +1 | |
| | 1 | 46 | | | | | +1 | | | −1 | +1 | −1 | |
| | 5 | 47 | | | | | | | | | | +1 | |
| | 1 | 47 | | | | | −1 | | | | | +2 | |
| | 1 | 47 | | | | | −1 | | | +1 | +2 | −1 | |
| | 1 | 47 | | | | | | | | +1 | −2 | +2 | |
| | 1 | 47 | | | | | | | | | +2 | −1 | |
| | 1 | 47 | | | | | | | | +2 | −1 | | |
| | 1 | 47 | | +1 | −1 | | +1 | −1 | | | | +1 | |
| | 1 | 48 | | | | | −1 | +1 | | +1 | | +1 | |
| | 1 | 48 | | | | | | | | +1 | +1 | | |

*Case* 5.—A.R., a 48-year-old man presented with a 6 months' history of progressive enlargement of the cervical and axillary lymph nodes and of the left tonsil. Material for chromosome analysis: lymph node; (a) only 2 metaphases had normal karyotype; (b) abnormal clone pseudodiploid in 10 cells (46, C—, D+, $E_{16}$+, G —); minimal deviations around this stem line karyotype in other 5 cells.

*Case* 6.—G.V.C., a 34-year-old woman was admitted for investigation of splenomegaly and pancytopenia. As no cause for her disease could be found, she was splenectomized, after which the peripheral blood returned to normal. She was readmitted 3 months later with enlargement of an axillary lymph node. Material for chromosome analysis: lymph node; (a) predominance of cells with normal karyotype; (b) abnormal clone hypertetraploid (92–96) with a large acrocentric marker chromosome.

*Case* 7.—A.C., a 56-year-old man was admitted with a 3 months' history of anaemia and hepatosplenomegaly. Bone marrow aspirates showed 50% of lymphosarcoma cells. Chromosome studies of this material showed: (a) predominantly normal metaphases; (b) an abnormal clone in 3 hypodiploid cells, containing 3 marker chromosomes, 1 large submetacentric and 2 large acrocentrics (43, # 1−, B−, C+, 5D−, 3E+, 2F−, 2 mar t), (44, C−, 5D−, $E_{16}$+, mar sm, 2 mar t), (45, B−, 5D−, 2G+, mar sm, 2 mar t). Severe karyotype changes must have occurred to originate this clone because only 1 D chromosome was recognized in each of the abnormal cells (Fig. 3).

*Case* 8.—M.B., a 62-year-old man was admitted for investigation of a retroperitoneal tumour and ascites. The symptoms could be traced back for 18 months. He showed extensive bone marrow infiltration. Chromosome preparations from lymph node cells were unsuccessful, and only 1 metaphase analysed showed normal karyotype. Many metaphases, with poor chromosome spreading, showed a large acrocentric marker which was easily identified by its peripheral situation in the metaphase plate. The ascitic fluid cells showed: (a) normal karyotype in 3 cells, and in 1 (48, C+, $E_{17-18}$+); (b) abnormal clone hypodiploid with a large acrocentric marker, 3 cells (44, 2C−, D−, $E_{17-18}$+, mar t) and small variations in 4 other cells.

*Case* 9.—J.A.N., a 62-year-old man presented with a 3 years' history of a slow but progressive generalized lymphadenopathy. On admission he was found to have a retroperitoneal tumour and abdominal visceral involvement. Material: lymph node; (a) normal karyotype in 10 metaphases; (b) 2 metaphases with identical karyotype (46, # 3−, B−, 2D−, G+, mar st, 2 mar t), probably represent an abnormal clone pseudodiploid containing 1 large subterminal and 2 large acrocentric marker chromosomes (Fig. 4). Chromosome preparations from bone marrow aspirates, which showed intense infiltration by lymphosarcoma cells, showed a normal karyotype in 9 metaphases, but abnormally large acrocentric chromosomes could be seen in some metaphases, in spite of poor chromosome spreading, occupying a peripheral position, as in the previous case.

### *Well differentiated lymphocytic lymphomas*

*Case* 10.—M.F., a 34-year-old man was admitted with generalized lymph node enlargement which had appeared 12 months previously. Material: lymph node; (a) normal karyotype in 1 metaphase with 46 and 1 with 92 chromosomes; (b) abnormal clone pseudodiploid with 2 markers, 1 subterminal and 1 submetacentric; 6 metaphases analysed showed inconsistent karyotypes, but followed a clonal relationship (Table II and Fig. 5).

*Case* 11.—G.R.L., a 47-year-old man had noticed generalized lymphadenopathy for 1 year. Material: lymph node; very few mitoses, 3 (46) normal karyotype, 1 (45, C−) and 1 (45, G−).

### *Reticulum cell sarcomas*

*Case* 12.—J.T., a 31-year-old woman was admitted because of loss of weight, diarrhoea and abdominal pain of 6 months' duration. She presented with ascites and a large palpable abdominal tumour. The sediment of ascitic fluid showed approximately 40% of malignant cells with numerous mitoses, mixed with mature lymphocytes. Chromosome analysis of this material showed: (a) normal karyotype in 24% of the cells; (b) a pseudodiploid clone confirmed by photographic analysis of 13 metaphases of identical karyotype where 2 group G chromosomes were substituted by 2 markers, one smaller than the members of group G and the other of size between that of D and G. The karyotype was otherwise apparently unchanged (Fig. 6). A possible mechanism of origin for these markers would be a long arm G/G reciprocal translocation, t(Gq+; Gq−); (c) a hyperdiploid clone (51 chromosomes) whose stem line karyotype was represented by 5 cells (51, 2 # 1 −, 2 # 2+, 2B+, 2C+, 3D+, F−, G−) (Fig. 7). Small variations around this karyotype were found in 9 other cells analysed. Normal cytology of the peripheral blood and bone marrow, and absence of $Ph_1$ chromosome in the bone marrow cells, excluded the diagnosis of chronic granulocytic leukaemia suggested by the presence of the $Ph_1$-like chromosome in one of the abnormal clones.

*Case* 13.—J.L.S., a 21-year-old man was admitted for investigation of weight loss and diarrhoea, and an abdominal tumour, which he had noticed 6 months before. Material for chromosome analysis: mesenteric lymph node; (a) normal karyotype in 10 metaphases; (b) 3 abnormal metaphases were found; they shared in common an increase of 1 group C chromosome and the other variations present were inconsistent (45, # 1−, C+, $E_{17-18}$+, 2F−), (46, C+, E16−), (46, # 3−, B+, C+, G−).

*Case* 14.—D.F., a 40-year-old man presented to hospital with a 15 months' history of pyrexia, weight loss and pallor. Ascites and a large retroperitoneal tumour were found on examination. Ascitic fluid was used for chromosome analysis. It showed 50% of abnormal reticulum cells and abundant mitoses. The analysis of 24 metaphases showed only 1 with normal karyotype and 5, with 47 chromosomes had identical karyotype where 2 small chromosomes with subterminal centromeres were slightly larger than the members of group G (Fig. 8). Variations around this karyotype were found in the other 18 metaphases analysed (Table II). Although the extra chromosomes were morphologically similar to a large Y, they could also be an altered No. 18 with a deletion in the long arm. Smears of ascitic fluid sediment were stained with quinacrine mustard and examined under fluorescence microscopy in order to identify the number of Y chromosomes present in the normal and malignant cells (Polani and Mutton, 1971). In the mature lymphocytes and in the polymorphonuclear leukocytes only 1 fluorescent spot was found, which is consistent with an XY somatic constitution. The abnormal reticulum cells showed no fluorescent spot in 64% of the cells, 1 in 28%, 2 in 8%, and occasional cells had 3. In comparison with the karyotype analysis, these results suggest that the karyotype in the malignant cells showed variable gains and losses of Y chromosomes, accompanied by other aneusomies, but the dominant clone presented gain of one Y.

The diagnosis of reticulum cell sarcoma in cases 12 and 14 were confirmed at post-mortem examination.

### *Hodgkin's Disease*

The distribution of chromosome counts is presented in Table III. Lymph node material was used for chromosome analysis in all cases.

*Lymphocyte predominance*

*Case* 15.—A.P.P., a 40-year-old man showed right cervical and supraclavicular lymphadenopathy, which had appeared 7 months previously. Chromosome analysis: only cells with normal karyotype were found.

*Case* 16.—W.C.S., a 30-year-old man was admitted complaining of right cervical lymph node enlargement for 15 months. Chromosome preparations showed only normal diploid cells.

*Case* 17.—P.B.F., a 41-year-old man was admitted with a 2½ years' history of cervical lymph node enlargement. He was treated with regional radiotherapy and achieved a remission lasting 8 months, following which he presented with

TABLE III.—*Distribution of Chromosome Counts in* 14 *Cases of Hodgkin's Disease*

| Case No. | No. of metaphases counted | Chromosome number 43 | 44 | 45 | 46 | 47 | 48 | More than 48 |
|---|---|---|---|---|---|---|---|---|
| Lymphocyte predominance | | | | | | | | |
| 15 | 46 | | | 2 | 44 | | | |
| 16 | 23 | | | 1 | 22 | | | |
| 17 | 50 | | 1 | 3 | 44 | 1 | | 1*(115) |
| Mixed cellularity | | | | | | | | |
| 18 | 63 | | | 3 | 54 | | | 1*(65) 1*(75) 1*(79) 3*(80) |
| 19 | 18 | | | | 15 | | | 1*(70) 1*(73) 1*(74) |
| 20 | 32 | | | 1 | 24 | | | 2*(66) 1*(67) 1*(68) 1*(69) 1*(70) 1*(71) |
| 21 | 44 | 2 | | 4 | 35 | | | 1*(57) 1*(72) 1(92) |
| 22 | 1 | | | | 1 | | | |
| Nodular sclerosis | | | | | | | | |
| 23 | 32 | | 1 | 1 | 18 | | | 1*(74) 1*(76) 1*(78) 8*(79) 1*(81) |
| 24 | 78 | 1 | | 5 | 45 | 1 | 1 | 1*(63) 1*(67) 1*(68) 1*(73) 1*(74) 3*(75) 4*(76) 1*(77) 2*(78) 3*(79) 2*(80) 1*(81) 1(91) 2(92) 1*(152) |
| 25 | 15 | 1 | | | 14 | | | |
| 26 | 3 | | | 1 | 2 | | | |
| 27 | 49 | | 2 | 1 | 36 | | | 1*(51) 1*(54) 1(65) 1(69) 1(72) 1*(89) 1*(93) 1*(95) 1*(99) 1*(100) |
| 28 | 2 | | | | 2 | | | |

* Represents the number of cells with marker chromosomes.

enlargement of the axillary lymph nodes. He was then treated with nitrogen mustard and cyclophosphamide. He was readmitted with generalized lymphadenopathy. Material for chromosome analysis: cervical lymph node; (a) normal karyotype in 98% of the metaphases; (b) only 1 hypertetraploid metaphase, 115 chromosomes, showed 1 large subterminal and 1 medium-sized subterminal markers (Fig. 9).

*Mixed cellularity*

*Case* 18.—J.L.B., a 29-year-old man was admitted with enlargement of right cervical lymph nodes which had appeared 8 months before. Chromosome analysis revealed: (a) normal karyotype in 90% of the metaphases; (b) abnormal clone hypertriploid (modal No. 80), from which 5 metaphases were analysed and showed small differences between them, but in each case a large submetacentric and a large acrocentric markers were seen (Fig. 10). In addition, a small minute chromosome was found in 3 metaphases and a dicentric in 1.

*Case* 19.—R.P., a 38-year-old man was admitted complaining of fever which had appeared 2 years before, followed by pruritus and cervical lymph node enlargement. Results of chromosome analysis: (a) normal karyotype in 83% of the metaphases; (b) all abnormal cells with 70, 73 and 74 chromosomes showed a very large submetacentric marker (Fig. 11).

*Case* 20.—J.R.A., a 40-year-old woman was admitted to hospital with a 7 months' history of cervical lymphadenopathy. Results of chromosome analysis: (a) normal karyotype in 78% of the metaphases; (b) abnormal clone, 66–71 chromosomes, from which 4 metaphases were analysed and revealed small karyotypic differences, but were characterized by 2–5 submetacentric and 2–3 subterminal markers (Fig. 12).

*Case* 21.—A.D.S., a 12-year-old boy was admitted because of right cervical lymphadenopathy which he had noticed 4 months previously. Chromosome analysis: (a) normal karyotype in 93% of the metaphases; (b) abnormal cells with 57 and 72 chromosomes showing a large acrocentric marker chromosome in both (57, 4 ≠ 2+, ≠ 3−, 4C+, 3D−, 3E−, 2F+, 6G+, 2 mar t), (72, 4 ≠ 2+, 4C +, D +, 5E +, 5F +, 6G +, mar t) (Fig. 13).

*Case* 22.—M.C., a 50-year-old man was admitted because of progressive loss of

---

EXPLANATION OF PLATES

FIG. 1.—Case 2. Karyotype of a metaphase with 47 chromosomes showing 2 markers, 1 large submetacentric and 1 small metacentric.

FIG. 2.—Case 3. Karyotype of a metaphase with 50 chromosomes. It has a large submetacentric marker.

FIG. 3.—Case 7. Karyotype of a hypodiploid metaphase (44) showing 1 submetacentric and 2 large acrocentric markers. Five group D chromosomes are missing.

FIG. 4.—Case 9. Karyotype of a pseudodiploid metaphase showing 1 large subterminal and 2 large acrocentric markers.

FIG. 5.—Case 10. Karyotype of a pseudodiploid metaphase from a well-differentiated lymphocytic lymphoma. It has 2 definite marker chromosomes, 1 subterminal and 1 submetacentric.

FIG. 6.—Case 12. Karyotype of a pseudodiploid metaphase showing a probable G/G reciprocal translocation producing an enlarged G and a $Ph_1$-like chromosome.

FIG. 7.—Case 12. Stem line karyotype of 1 abnormal clone (51 chromosomes). There is no chromosome which can be identified as No. 1.

FIG. 8.—Case 14. Stem line karyotype (47 chromosomes), showing 2 chromosomes (arrows) morphologically similar to a large Y.

FIG. 9.—Case 17. Karyotype of a metaphase with 115 chromosomes. It has 2 subterminal markers of different size.

FIG. 10.—Case 18. Karyotype of a hypertriploid metaphase (79) showing a large submetacentric and a large acrocentric marker chromosomes.

FIG. 11.—Case 19. Karyotype of a metaphase with 74 chromosomes. It shows a very large submetacentric marker.

FIG. 12.—Case 20. Karyotype of a metaphase with 70 chromosomes. It has 3 large submetacentric and 2 medium-sized subterminal markers.

FIG. 13.—Case 21. Karyotype of a metaphase with 72 chromosomes showing a large acrocentric marker chromosome.

FIG. 14.—Case 23. Karyotype of a metaphase of the abnormal clone; 79 chromosomes, 2 minutes and a subterminal marker.

FIG. 15.—Case 24. Karyotype representative of the hypertriploid clone (76 chromosomes) showing 3 acrocentric marker chromosomes (arrows).

FIG. 16.—Case 27. Karyotype of a metaphase with 89 chromosomes. It has a giant subterminal marker chromosome, probably dicentric, and 2 large subterminal markers. The arrows point a triradial interchange and an acentric fragment.

1

2

Coutinho, Bottura and Falcao.

3

4

A1
A2
A3
B4-5
C6, X-12
D13-15
E16
E17
E18
F19-20
G21-22, Y
st sm

5

A1
A2
A3
B4-5
C6, X-12
D13-15
E16
E17-18
F19-20
G/G G21-22

6

7

8

Coutinho, Bottura and Falcao.

9

10

Coutinho, Bottura and Falcao.

11

12

Coutinho, Bottura and Falcao.

13

14

15

16

Coutinho, Bottura and Falcao.

weight and fever for 9 months. There was radiological evidence of mediastinal enlargement. A pre-scalene lymph node biopsy was done. The resulting material was unsuitable for chromosome analysis; only 1 metaphase with normal karyotype was found.

*Nodular sclerosis*

*Case* 23.—L.M.B., a 24-year-old man had noticed pain in the right groin for 2 months, and a palpable mass appeared 15 days before admission. Results of chromosome analysis: (a) normal karyotype in 62% of the metaphases; (b) abnormal clone hypertriploid (modal No. 79), from which 9 metaphases were analysed showing small variations between them, including 1–2 large subterminal marker chromosomes and 1–2 minute chromosomes (Fig. 14).

*Case* 24.—M.L.C., a 26-year-old woman was admitted with a 6 months' history of cough, loss of weight, lymphadenopathy and radiological evidence of mediastinal enlargement. Chromosome analysis: (a) normal karyotype in 66% of the metaphases; (b) abnormal clone hypertriploid (modal No. 76), 12 metaphases were analysed showing an inconsistent karyotype, but which followed a clonal pattern, which included 2–3 acrocentric markers slightly larger than the members of group D (Fig. 15).

*Case* 25.—A.D., a 19-year-old man was admitted to hospital with a history of bilateral cervical lymphadenopathy which had appeared 18 months before. Cytogenetic analysis revealed only metaphases with normal diploid karyotype.

*Case* 26.—A.O., a 27-year-old woman was admitted with a 5 months' history of cervical lymph node enlargement, cough and chest pain. Radiological examination revealed a probable parenchymal infiltration of the left lung. Sparse mitoses were found in the chromosome preparations. Only 2 metaphases were analysed and showed normal karyotype.

*Case* 27.—F.B.C., a 17-year-old man was admitted with a 6 months' history of night sweats and cervical lymphadenopathy. There was radiological evidence of mediastinal enlargement. The liver was palpable 2 cm. below the costal margin and the spleen 5 cm. Haematological investigation showed that he suffered also from thalassaemia trait. Material for chromosome analysis was a cervical lymph node: (a) normal karyotype in 80% of the metaphases; (b) there were apparently 3 abnormal stem lines. The cells with 65, 69 and 72 chromosomes had similar karyotype changes and probably represent one abnormal clone. No marker was found in these cells (69, # 2+, # 3+, 2B+, 7C+, 3D+, $4E_{16}+$, $E_{17-18}-$, 4F+, 2G+). Both cells with 51 and 54 chromosomes showed identical subterminal markers and decrease of group G chromosomes, and probably form a second abnormal clone. The hypertetraploid cells (89–100 chromosomes) apparently formed a clone which was double the last one (Fig. 16). The subterminal markers were duplicated, and in the evolution, these cells gained one giant subterminal marker which was found in 2 metaphases and, in a third, it was represented by a large ring and 2 large acentric fragments.

*Case* 28.—P.P.S., a 49-year-old man gave a 4 months history of weakness, pallor and cervical lymphadenopathy. The liver was palpable 4 cm. below the costal margin and the spleen 5 cm. The material for chromosome analysis was taken from a cervical lymph node which appeared partly necrotic and resulted in poor chromosome preparations. Only 2 metaphases were analysed, with normal diploid karyotype.

### DISCUSSION

A survey of the previous literature by the present authors shows that chromosome studies in malignant lymphomas were infrequently performed. Considering together isolated cases and relatively small series, 19 cases of lymphoblastic lymphosarcoma, 7 of lymphocytic lymphosarcoma, 24 of reticulum cell sarcoma, 9 of follicular lymphoma and 10 of Burkitt's tumour have so far been reported (Tjio *et al.*, 1963; Jacobs *et al.*, 1963; Sandberg *et al.*, 1964; Baker and Atkin, 1965; Fitzgerald and Adams, 1965; Sasaki *et al.*, 1965; Stewart *et al.*, 1965; Atkin *et al.*, 1966; Miles *et al.*, 1966; Miles, 1967; Kajii *et al.*, 1968; Lawler *et al.*, 1968; Millard, 1968; Spiers and Baikie, 1968; Clarkson *et al.*, 1969). In a review of Hodgkin's disease by Seif and Spriggs (1967), they collected 22 observations including 8 of their own. Since then, only 5 more cases have been reported (Millard, 1968; Spiers and Baikie, 1968). The techniques which have been employed were either direct method or short term cultures. A direct method of chromosome analysis is indispensable for karyotyping tumour cells although some authors have claimed that better chromosome preparations are obtained after short periods of culture, varying from 12 to 72 hours (Seif and Spriggs, 1967; Spiers and Baikie, 1968). The comparison between the cases previously reported is not an easy task because large numbers of them were incompletely documented with the result that one cannot determine what was the abnormal karyotype. Two obstacles are mainly to blame for this. Firstly, the dividing malignant cells may not have been plentiful, and this applies mainly to Hodgkin's disease. The second is the difficulty in obtaining good chromosome preparations from direct sampling of cells of solid tumours. When suitable material is available, chromosome abnormalities are found in approximately 80% of cases.

In this series, chromosome abnormalities were found in 12 of the 14 cases of lymphocytic lymphomas and reticulum cell sarcomas, and in 8 of the 14 cases of Hodgkin's disease. The chromosome abnormalities were both numerical and structural and were characterized by clones of cells with abnormal karyotypes. The number of chromosomes of the abnormal cells was near-diploid in the lymphocytic lymphomas and reticulum cell sarcomas, except one case which was hypertetraploid, while in Hodgkin's disease it was in the hypertriploid range. In individual cases the karyotype was seldom homogeneous as it is in the normal tissues, but a clonal pattern was recognized in every abnormal cell line, indicating a common precursor for these cells. The karyotype diversity was due to small excesses or losses of chromosomes in different groups. In the majority of cases of lymphocytic lymphomas a homogeneous dominant karyotype (stem line) was found, with the accompanying cohort of related cells with small variations around the basic karyotype. These cells are probably generated by secondary mitotic errors in the already abnormal stem line. This cytogenetic instability within the tumour cells is a characteristic of malignant proliferation and can make the basis for clonal evolution. On the other hand, the aberrant cells resulting from these variations may be less successful and responsible for either the slow proliferating or for the non-proliferating pool, or for the cell deaths in the tumour (Killman, 1968).

The comparison between the abnormal clones of different cases revealed that each tumour carries its specific stem line which is distinct from the others, even of the same histologic type. No karyotype abnormality was found to be specifically related to the disease. All chromosome groups were involved and the changes did not follow a common pattern. Abnormalities of chromosomes of group E, mainly No. 18, were reported to be present in some cases of malignant lymphomas. Deletions of the short arm were described in 4 cases (Kajii *et al.*, 1968; Spiers and Baikie, 1968), and of the long arm in 6 cases (Seif and Spriggs, 1967; Millard, 1968). This subject was reviewed by Spiers and Baikie (1970). Accentuation of secondary constrictions of $C_9$ chromosomes were also described in malignant lymphomas (Miles *et al.*, 1966). This feature was not observed in the present series.

The marker chromosomes found showed varied morphology although large acrocentric markers were found in 7 of the 20 abnormal stem lines here reported. It was single, double or triple and appeared either alone or associated with other markers. Morphologically similar chromosomes were reported in other malignant lymphomas (Sandberg *et al.*, 1964; Sasaki *et al.*, 1965; Seif and Spriggs, 1967; Lawler *et al.*, 1968), Burkitt's tumour (Jacobs *et al.*, 1963; Stewart *et al.*, 1965), multiple myeloma and acute leukaemias (Tassoni *et al.*, 1967). To these cases can be added 3 more cases of multiple myeloma and one of acute lymphoblastic leukaemia studied by the present authors. Whether this marker is more frequently associated with these lymphoproliferative disorders or it is responsible for some common characteristic of the disease cannot be concluded from the available data. Large acrocentric chromosomes were also found in abnormal clones of carcinomas of the ovary, of the oesophagus, and of the bile duct (Coutinho, 1968).

The chromosome findings in Hodgkin's disease show two interesting features which distinguish them from the other lymphomas. First there is, in the majority of cases, a double population; a predominant one which shows a normal karyotype and a second, less conspicuous, showing chromosomal abnormalities following a clonal pattern. The findings confirm the idea that Hodgkin's disease is a neoplastic disease, but there is no cytogenetic evidence to support the concept that the lymphocyte population is also neoplastic. We share the opinion of Seif and Spriggs (1967) that the abnormal karyotype belongs to the abnormal reticulum cells and these represent the malignant cells of the disease. The normal metaphases belong to the histiocytes and plasma cells, which can represent an immune or inflammatory reaction. In view of the difficulty in recognizing to which cytological category a metaphase plate belongs, after the treatment for chromosome preparations, there is not yet conclusive evidence in favour of this hypothesis. The second feature is that the number of chromosomes of the abnormal clones is mainly in the hypertriploid range (69–80 chromosomes), with rare exceptions. It is unusual to find metaphases of higher ploidy. According to Atkin *et al.* (1966) there is, in tumours, a good correlation between the chromosome number and the modal DNA value of interphase cells, when estimated by Feulgen microspectrophotometry. Consequently, the chromosome numbers of the abnormal cells in Hodgkin's disease should correspond to a modal DNA value in the 3-3·5 n range. This expected figure is in disagreement with the work of Petrakis *et al.* (1959) who found diploid and tetraploid DNA values in Reed–Sternberg cells. They also found markedly increased DNA values, in the polyploid range, in multinucleated Reed–Sternberg cells. Certainly, if these multinucleated cells were proliferating, much higher numbers of chromosomes would be found. It can be concluded from this discussion that the proliferating pool of malignant cells in Hodgkin's disease consists of mononuclear abnormal reticulum cells and not the large multinucleated Reed–Sternberg cells. This conclusion is corroborated by the observation of the rarity of mitotic figures, in these polyploid cells, in histologic preparations.

The chromosome findings in the malignant lymphomas did not help to clarify controversial points in relation of the classification of the different histologic types, but can be a valuable tool to establish the differential diagnosis between malignant proliferation and reactive lymphoid hyperplasia in doubtful cases. In fact, no karyotypic abnormalities were found, in lymph nodes with benign enlargement, in 12 cases reported by Baker and Atkin (1965), in 9 by Seif and Spriggs (1967), and in 3 by the present authors; but Caprio *et al.* (1966) described, in the lymph node of a patient with non-specific lymphadenopathy, variable aneuploidy and metaphases with 47 chromosomes including 1 extra small acrocentric. The abnormalities were related to a probable viral aetiology although they could also be an expression of a pre-neoplastic change. A long-term follow-up would have proved whether these karyotypic alterations were part of an evolving neoplastic process, but no follow-up evidence was presented. Furthermore, the cytogenetic studies in the malignant lymphomas did not show any peculiarity which could differentiate them from the lymphocytic leukaemias. Numerical and structural chromosome abnormalities were found in both diseases, with the exception of the chronic lymphocytic leukaemias. In fact, no chromosome abnormality has been related so far to this disease, other than the familial association of a deletion in the short arm of a group G chromosome ($Ch_1$) and aneuploidy in culture, not confirmed by others (Lawler *et al.*, 1968).

Intense cytogenetic changes should have occurred in the genesis or evolution of these neoplasias in order to produce such abnormal chromosome combinations. Non-disjunction and " lagging ", rather simple mechanisms, can account for the aneusomies seen, but from the presence of marker chromosomes one can infer that major recombinations of the genetic material have occurred.

We would like to thank Dr. Dimitra Anagnostou, Honorary Research Assistant of the Department of Morbid Anatomy, Royal Postgraduate Medical School, for her help with the histological diagnosis, Mr. David Mutton, of the Pediatric Research Unit, Guy's Hospital Medical School, for his help with the quinacrine mustard staining, and Miss Marly H. Tavela, for her expert technical help.

This work has been partially supported by a grant from the " Fundação de Amparo a Pesquisa do Estado de São Paulo ", Brazil.

### REFERENCES

ATKIN, N. B., MATTINSON, G. AND BAKER, M. C.—(1966) *Br. J. Cancer*, **20**, 87.

BAKER, M. C. AND ATKIN, N. B.—(1965) *Br. med. J.*, i, 770.

CAPRIO, G., NESPOLO, A. AND BONADONNA, G.—(1966) *Tumori*, **52**, 433.

CHICAGO CONFERENCE.—(1966) Birth Defects: original article series, II.2. N.Y. (The National Foundation).

CLARKSON, B. D., THORNBECKE, G. J., HARVEN, E. AND MILES, C.—(1969) *Cancer Res.*, **29**, 823.

COUTINHO, V.—(1968) ' Chromosome studies in neoplasia: lymphomas and carcinomas with a special reference to the polyploidy by endoreduplication '. Thesis. Faculty of Medicine of Ribeirao Preto, University of São Paulo, Brazil.

FITZGERALD, P. H. AND ADAMS, A.—(1965) *J. natn. Cancer Inst.*, **34**, 827.

JACOBS, P. A., TOUGH, I. M. AND WRIGHT, D. H.—(1963) *Lancet*, ii, 1144.

KAJII, T., NEU, R. L. AND GARDNER, L. I.—(1968) *Cancer, N.Y.*, **22**, 218.
KILLMAN, T. S.—(1968) *Ser. Haematol.*, **1**, 38.
LAWLER, S. D., PENTYCROSS, C. R. AND REEVES, B. R.—(1968) *Br. med. J.*, iv, 213.
LEVAN, A., FREDGA, K. AND SANDBERG, A. A.—(1964) *Hereditas*, **52**, 201.
LUKES, R. J. AND BUTLER, J. J.—(1966) *Cancer Res.*, **26**, 1063.
MILES, C. P.—(1967) *Cancer, N.Y.*, **20**, 1253.
MILES, C. P., GELLER, W. AND O'NEILL, F.—(1966) *Cancer, N.Y.*, **19**, 1103.
MILLARD, R. E.—(1968) *Eur. J. Cancer*, **4**, 97.
PETRAKIS, N. L., BOSTICK, W. L. AND SIEGEL, B. V.—(1959) *J. natn. Cancer Inst.*, **22**, 551.
POLANI, P. E. and MUTTON. D. E.—(1971) *Br. med. J.*, i, 138.
RAPPAPORT, H.—(1966) ' Atlas of Tumor Pathology. Tumors of the hematopoietic system '. Armed Forces Institute of Pathology, National Academy of Sciences, Washington, D.C.
SANDBERG, A. A., ISHIHARA, T., KIKUCHI, Y. AND CROSSWHITE, L. H.—(1964) *Cancer, N.Y.*, **17**, 738.
SASAKI, M. S., SOFUNI, T. AND MAKINO, S.—(1965) *Cancer, N.Y.*, **18**, 1007.
SEIF, G. S. F. AND SPRIGGS, A. I.—(1967) *J. natn. Cancer Inst.*, **39**, 557.
SPIERS, A. S. D. AND BAIKIE, A. G.—(1968) *Cancer, N.Y.*, **22**, 193.—(1970) *Br. J. Cancer*, **24**, 77.
STEWART, S. E., LOVELACE, E., WHANG, J. AND NGU, V. A.—(1965) *J. natn. Cancer Inst.*, **34**, 319.
TASSONI, E. M., DURANT, J. R., BECKER, S. AND KRAVITZ, B.—(1967) *Cancer Res.*, **27**, 806.
TJIO, J. H., MARSH, J. C., WHANG, J. J. AND FREI III, E.—(1963) *Blood*, **22**, 178.


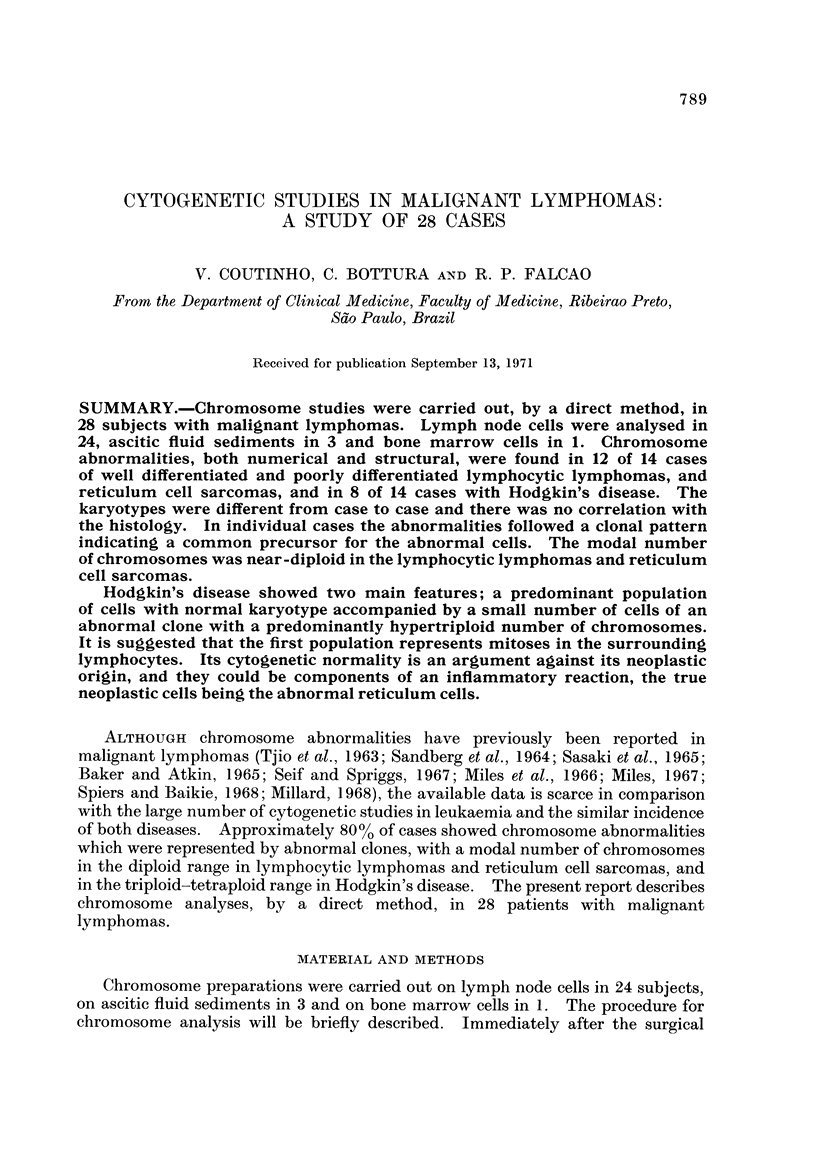

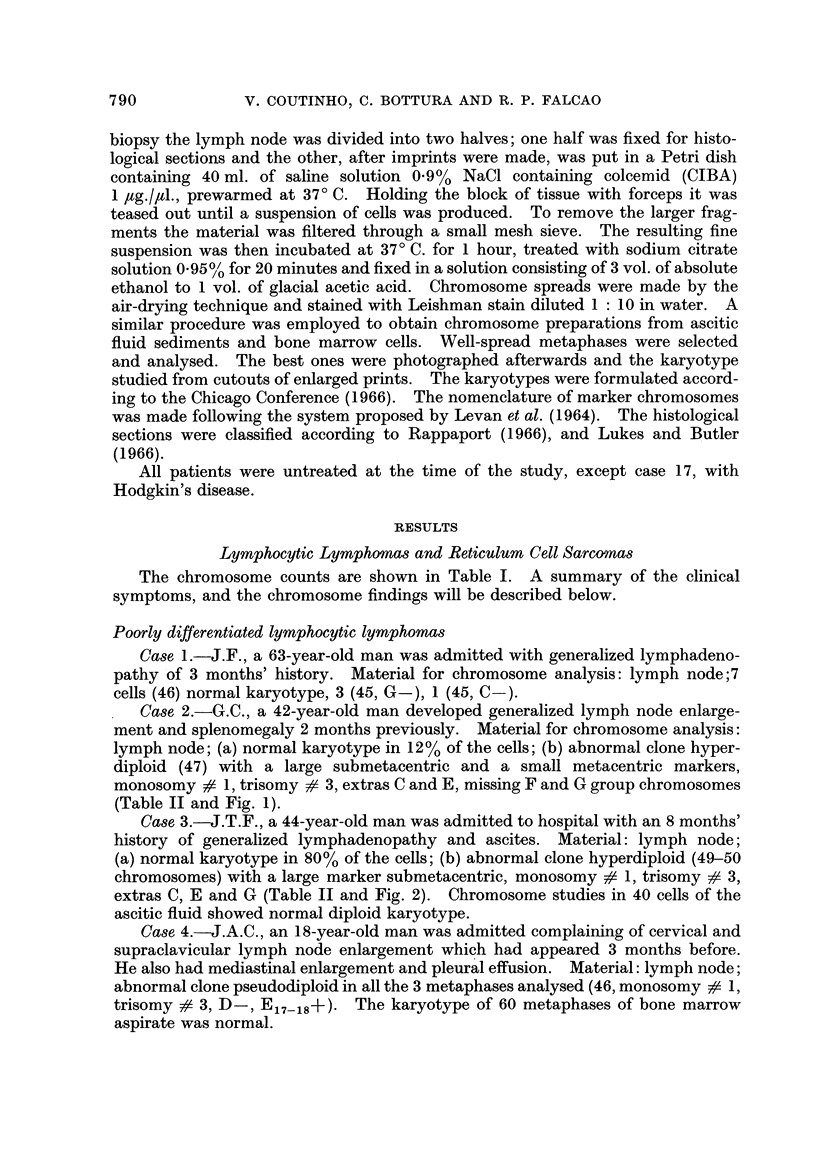

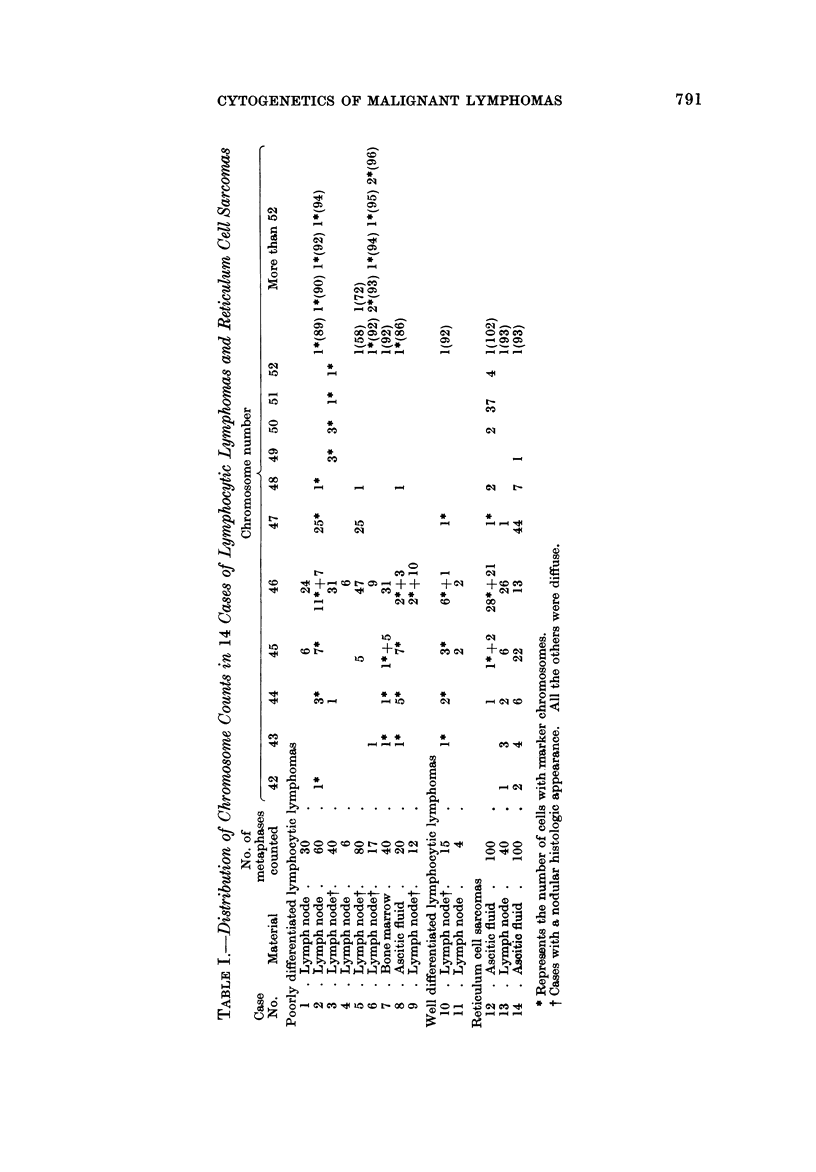

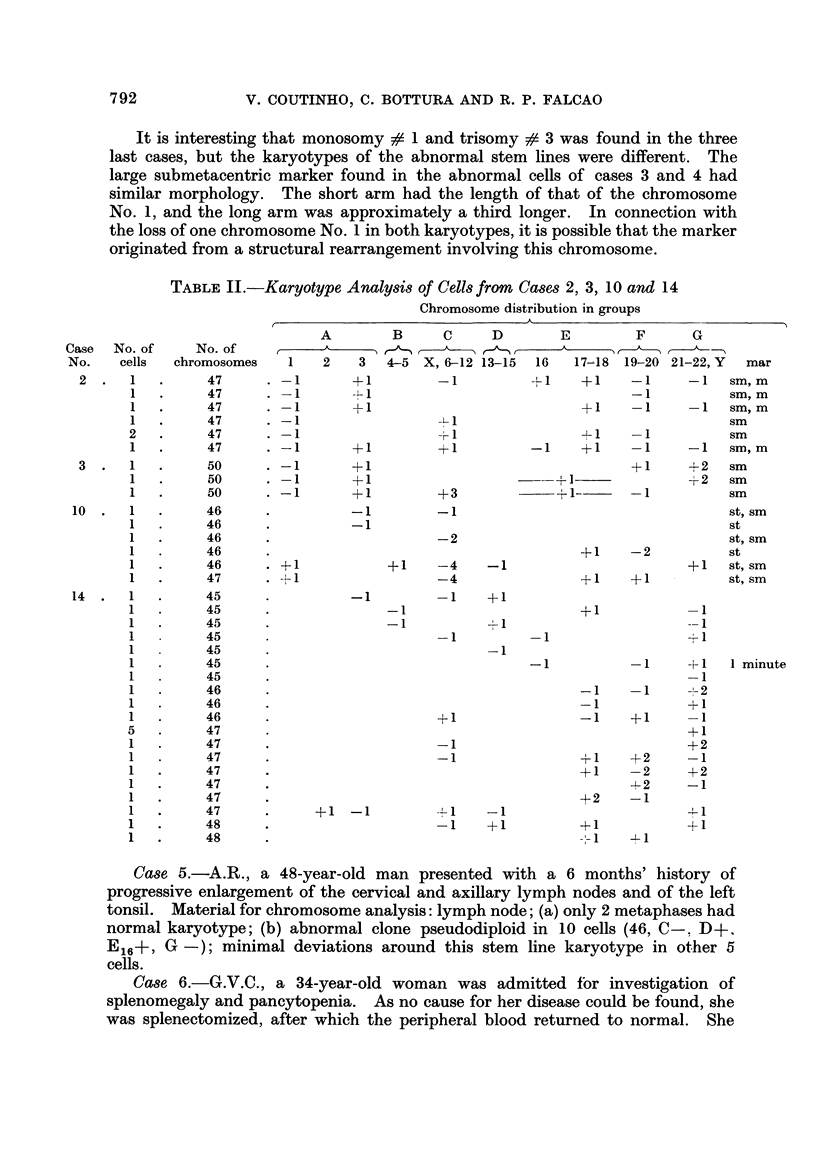

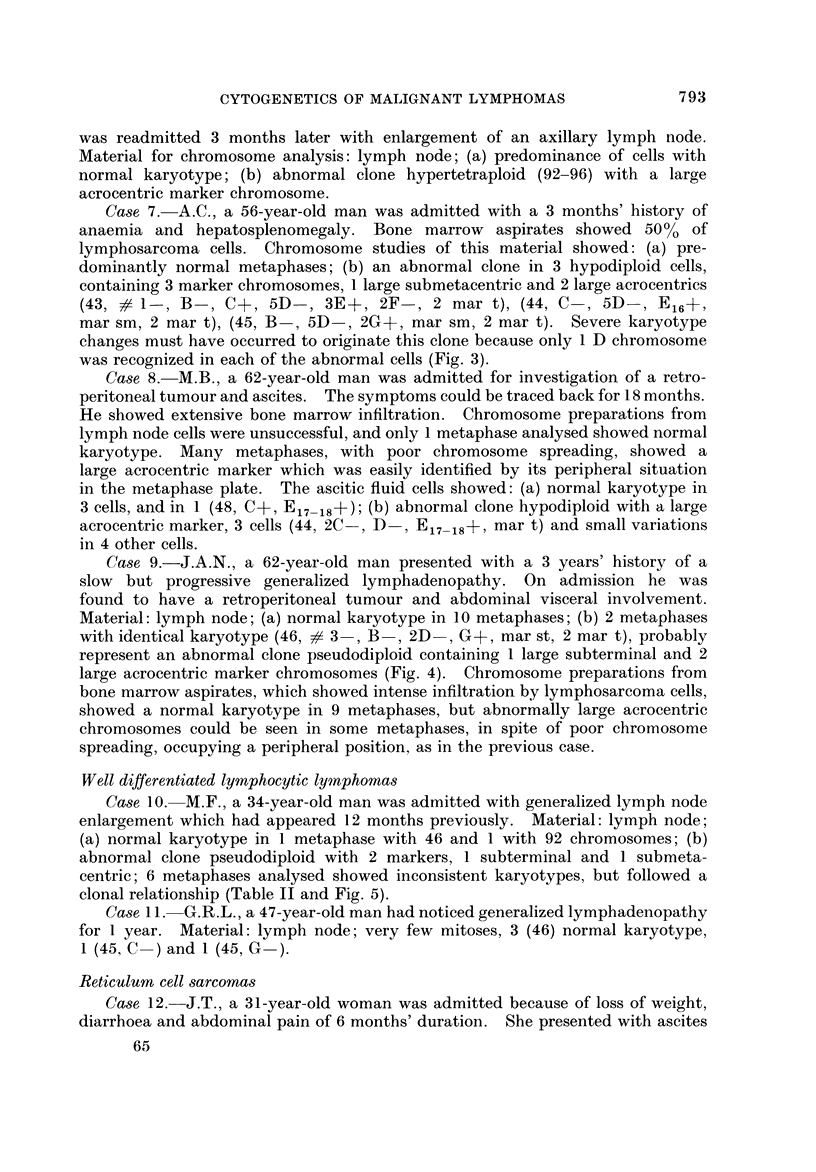

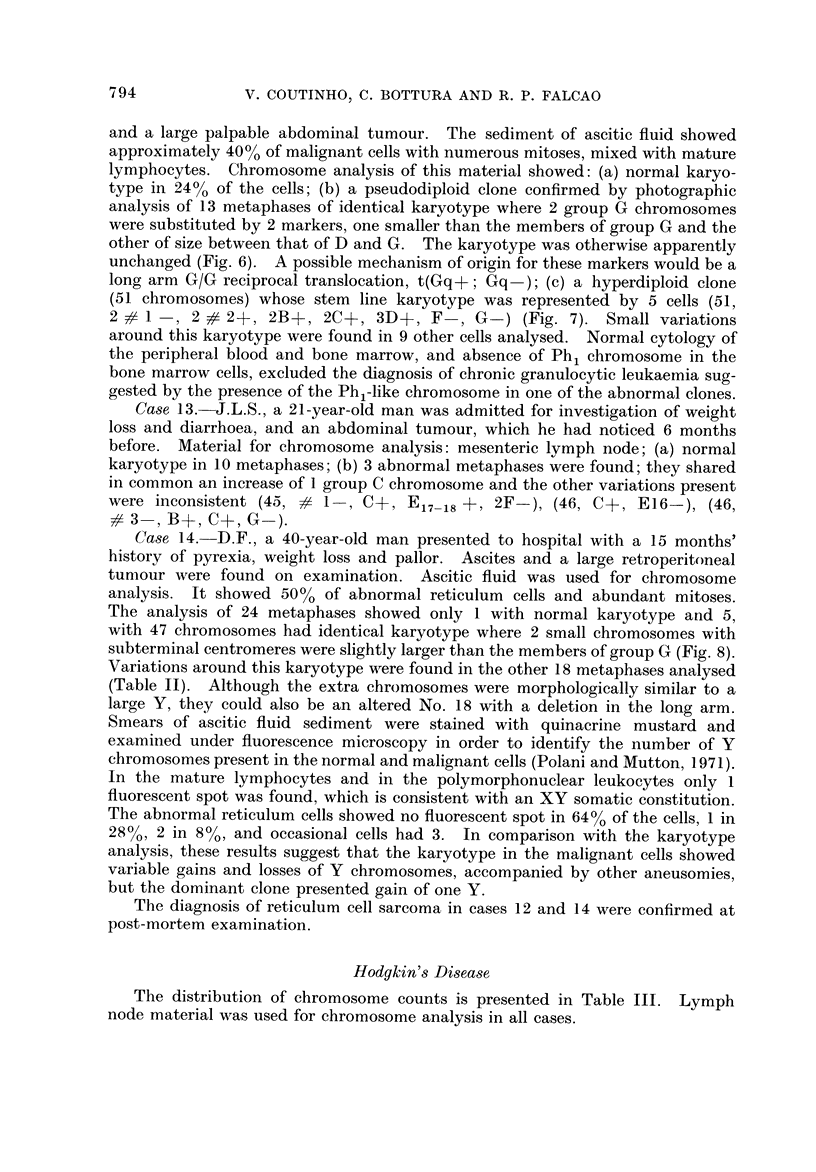

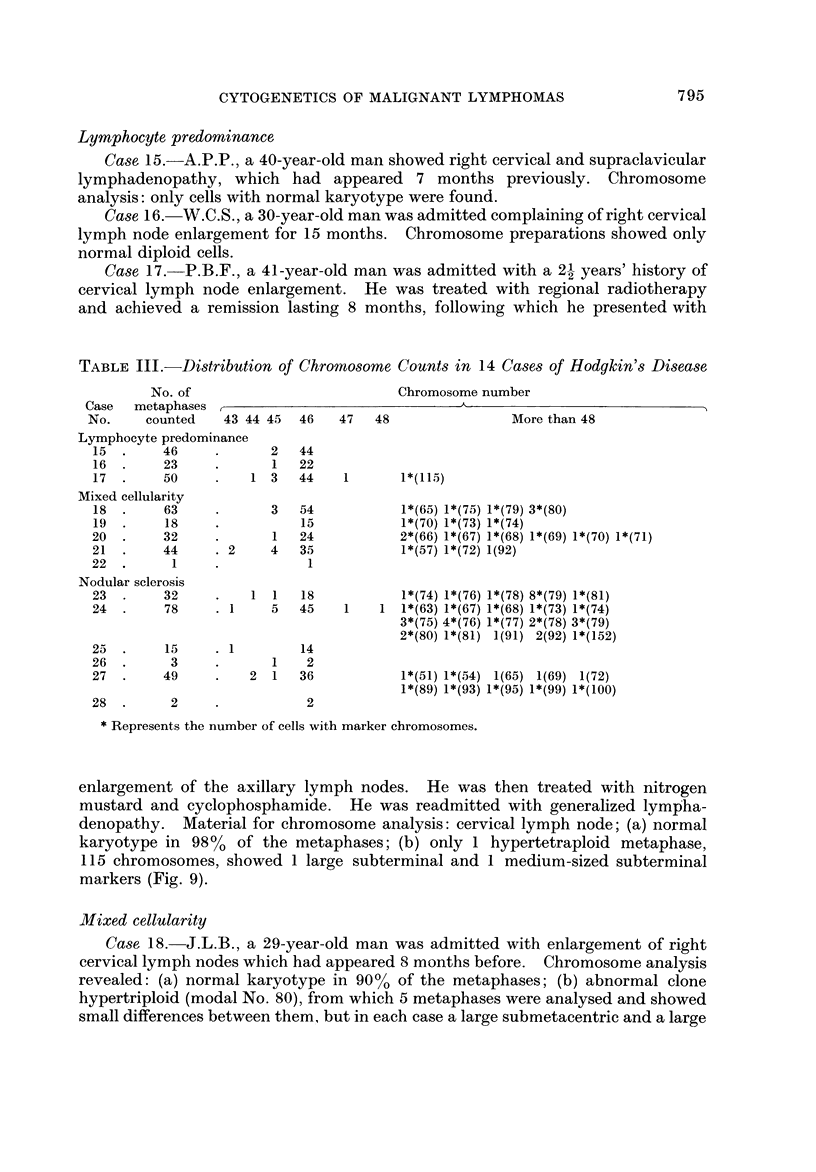

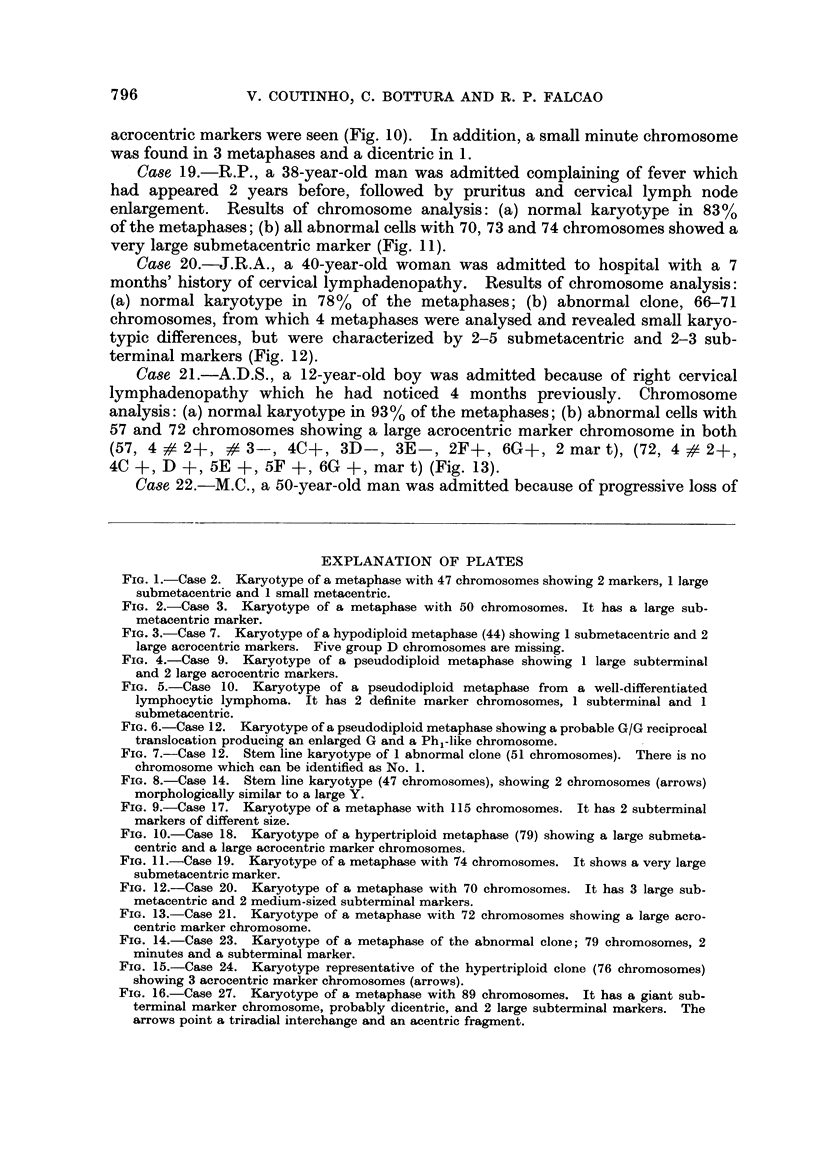

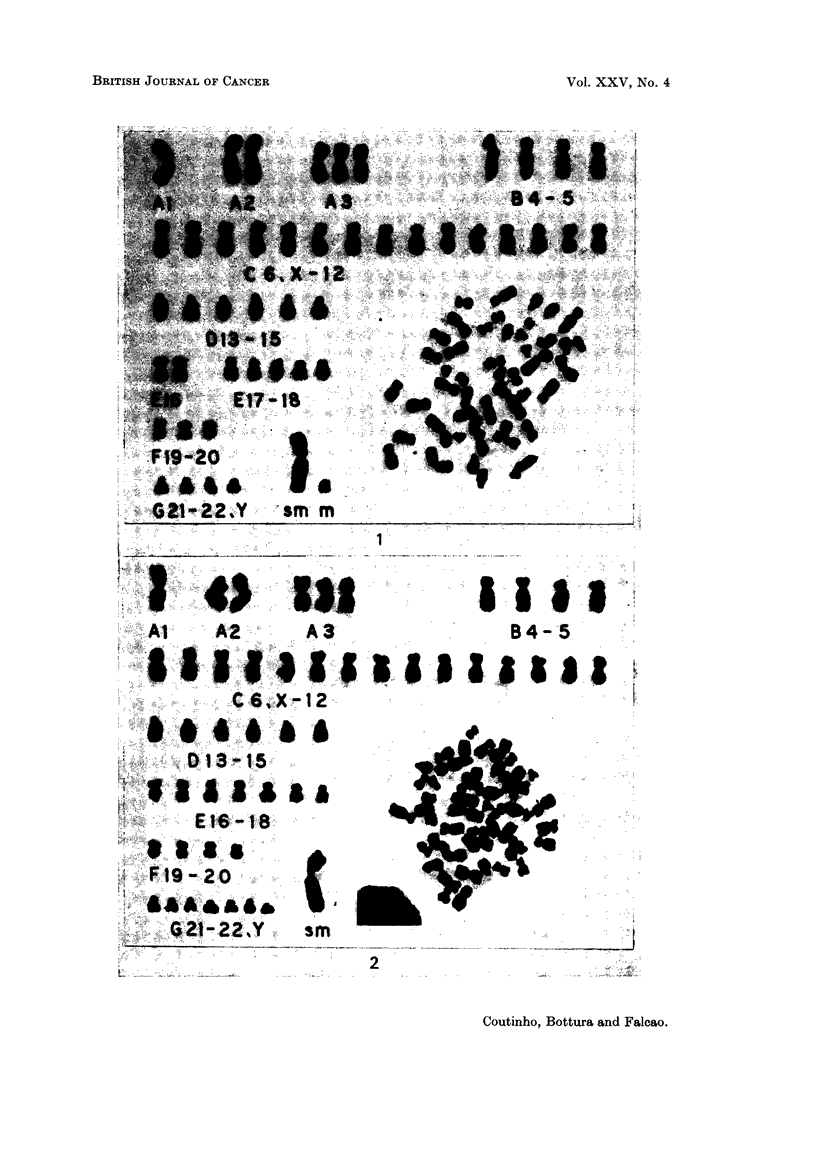

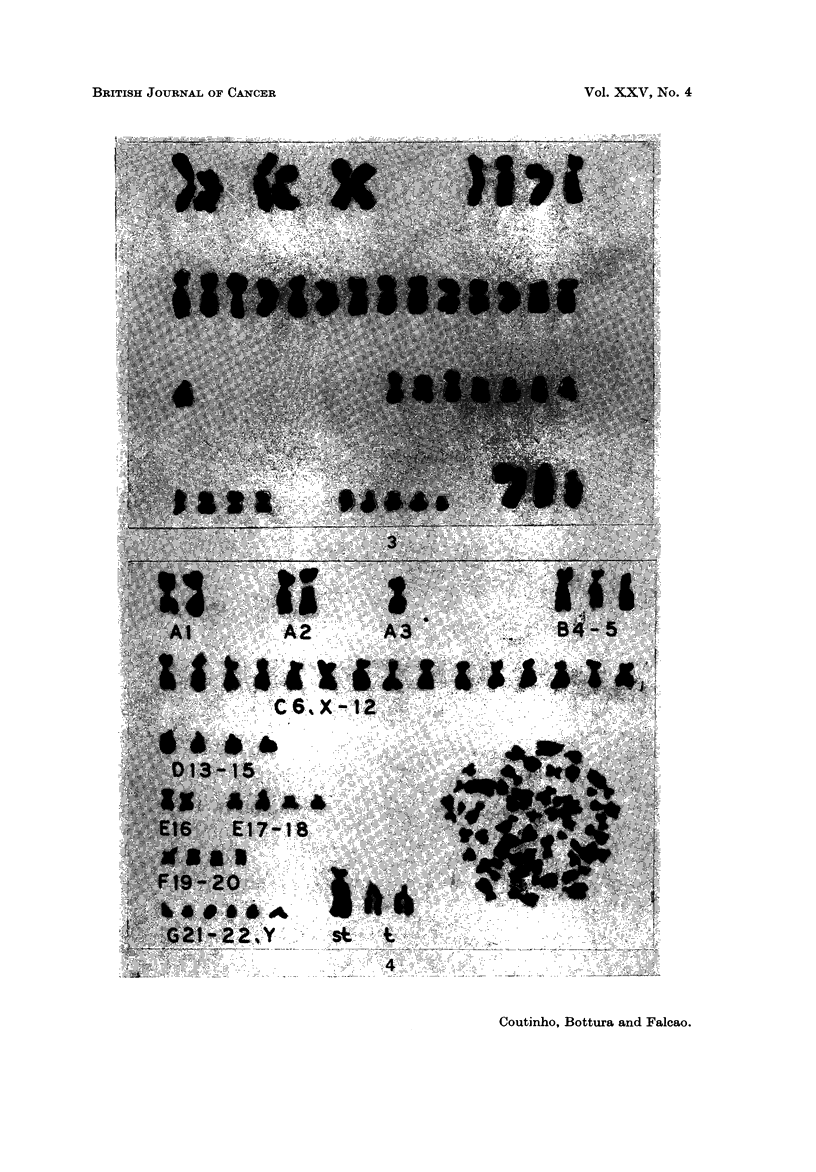

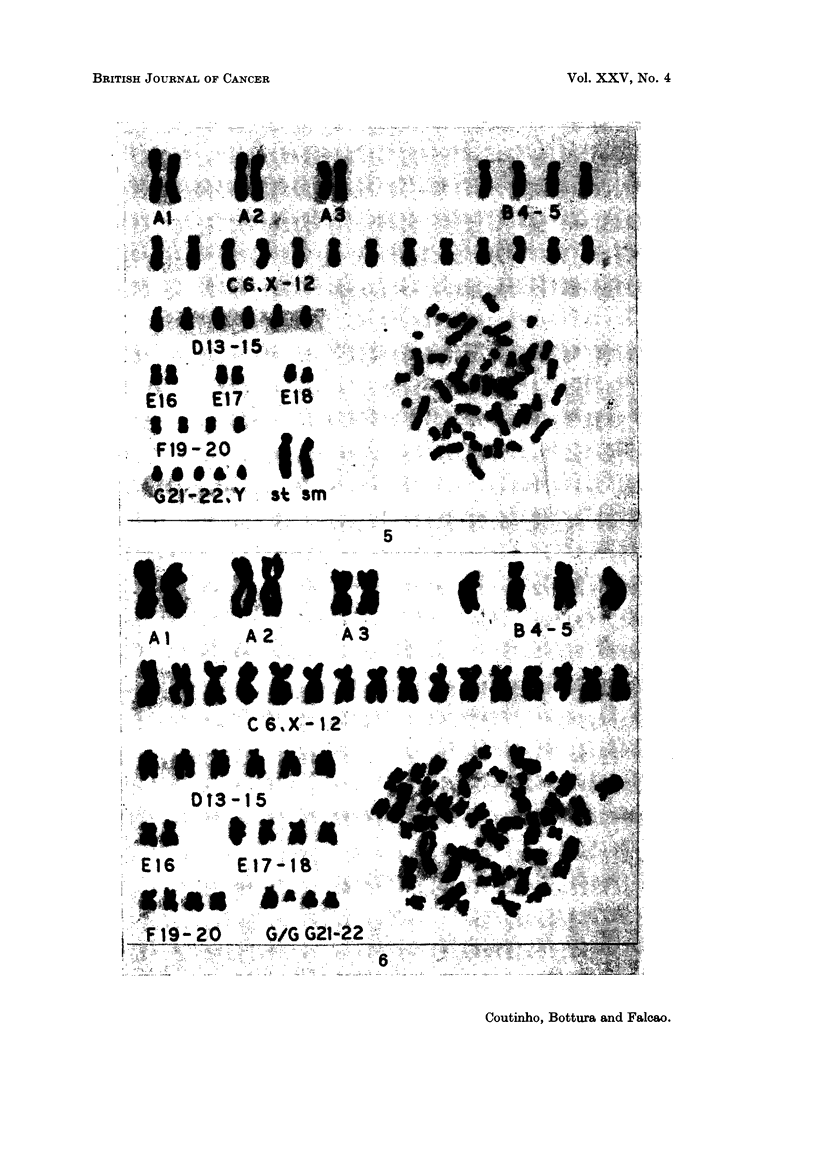

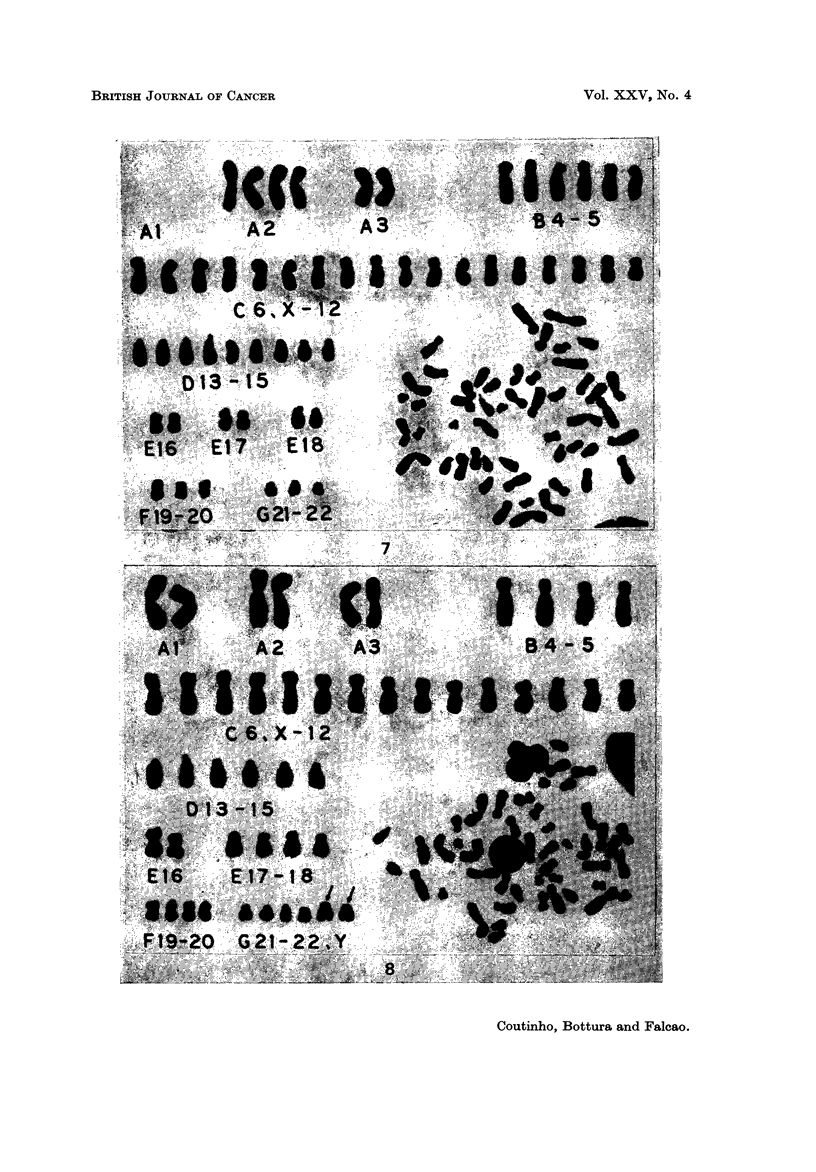

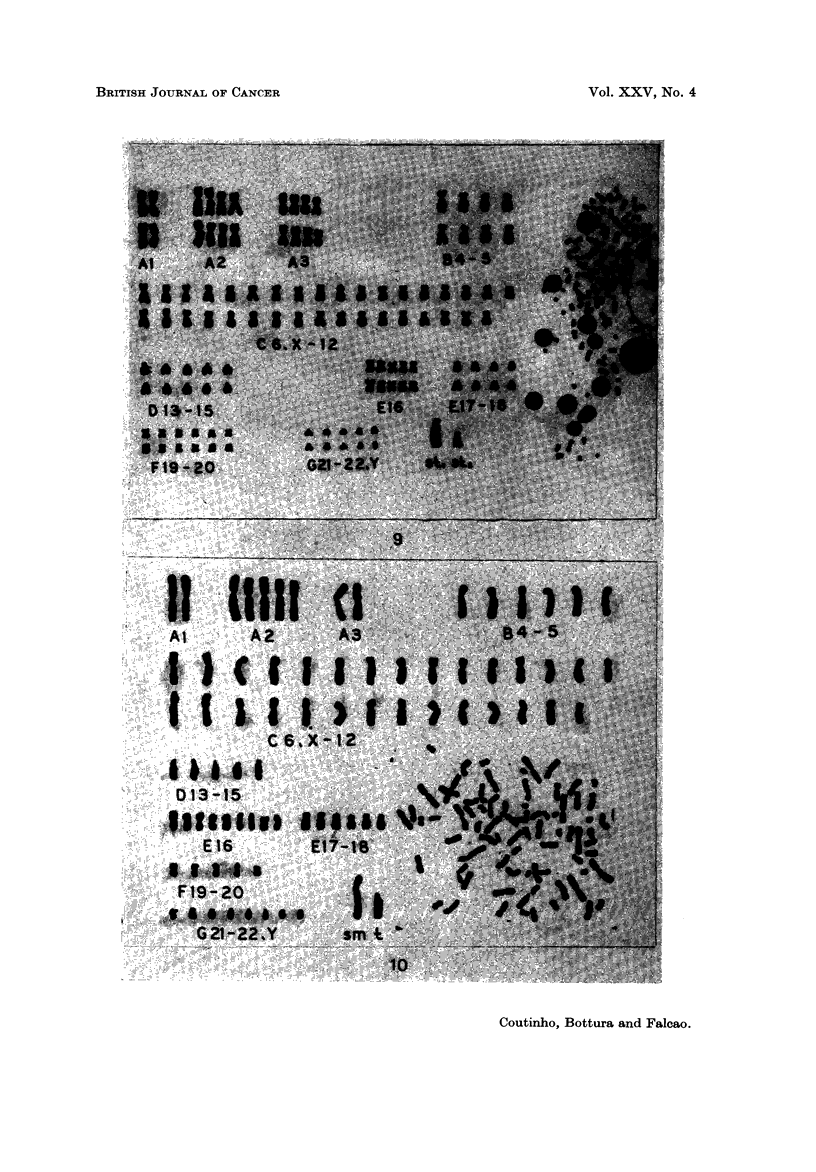

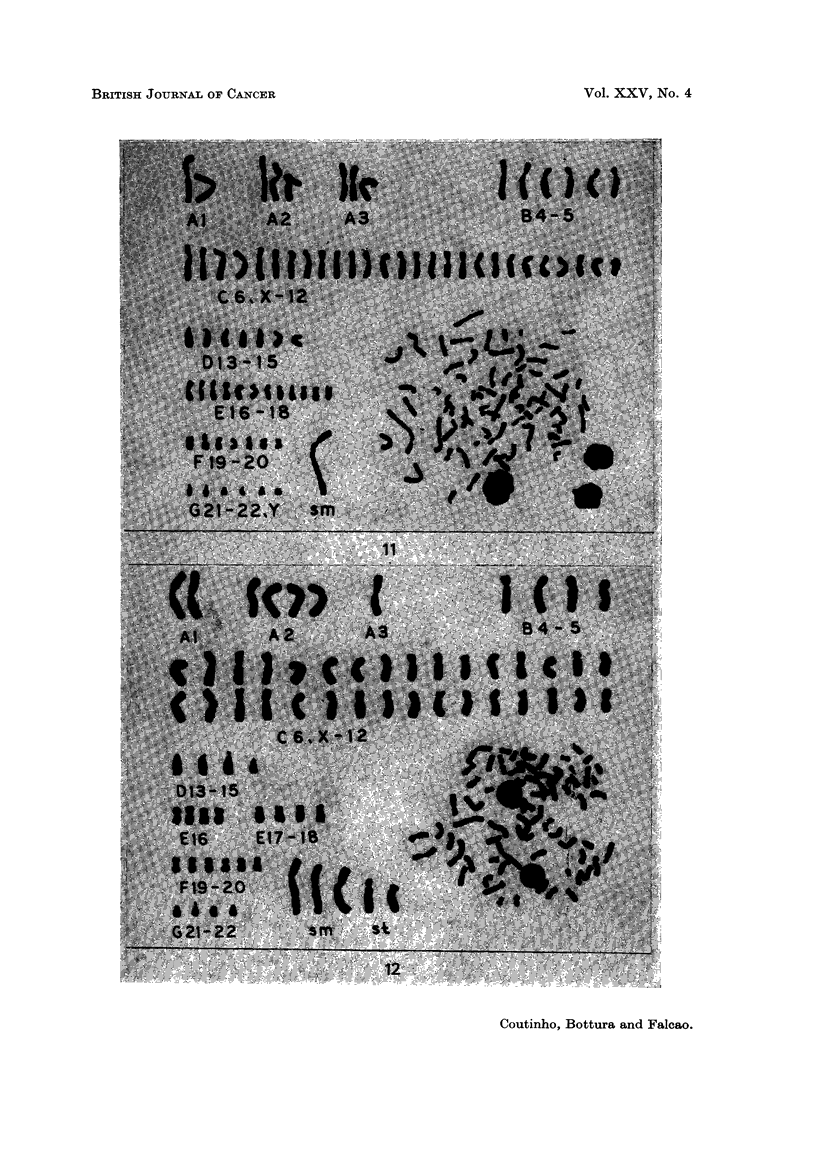

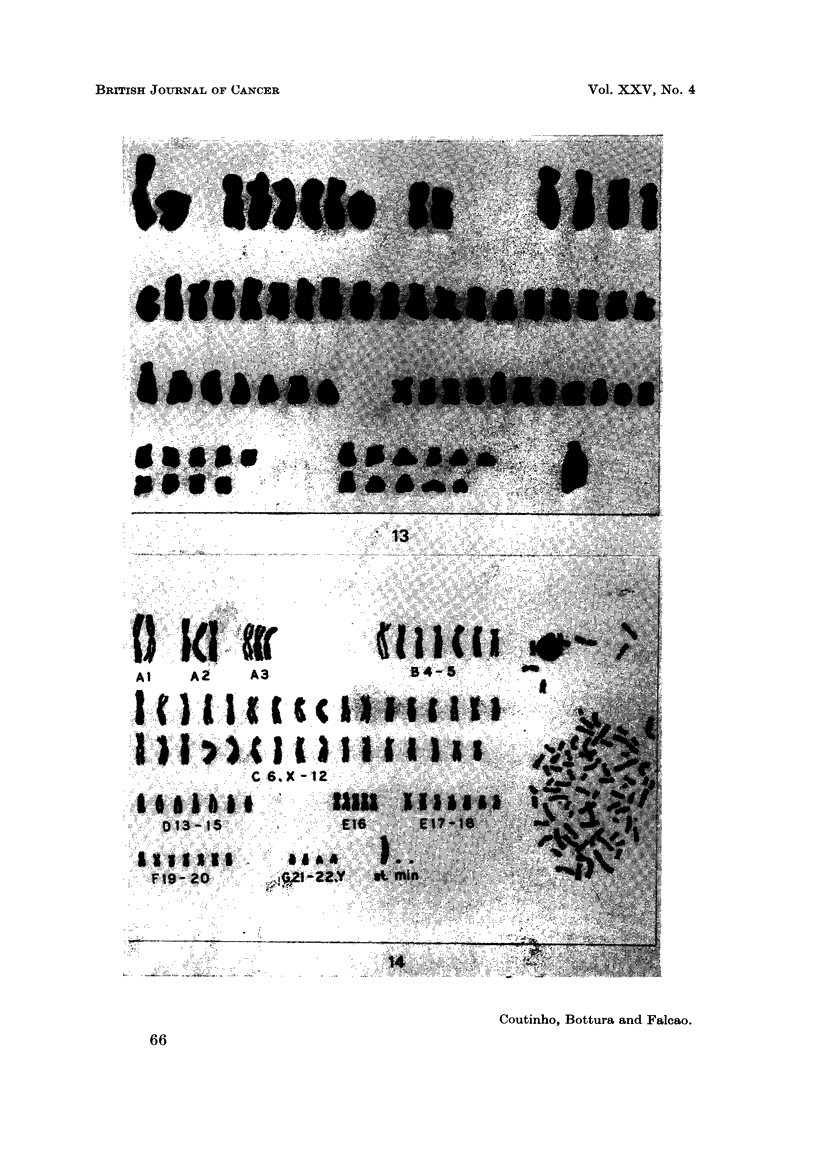

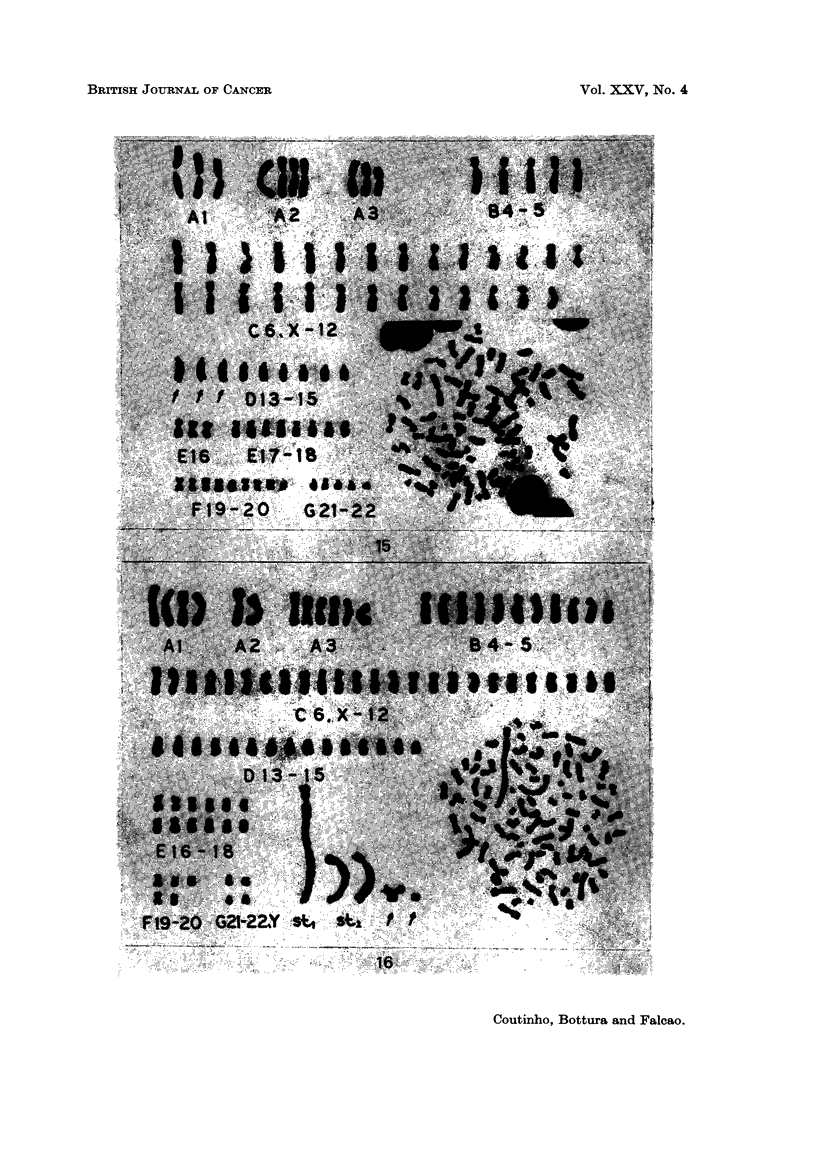

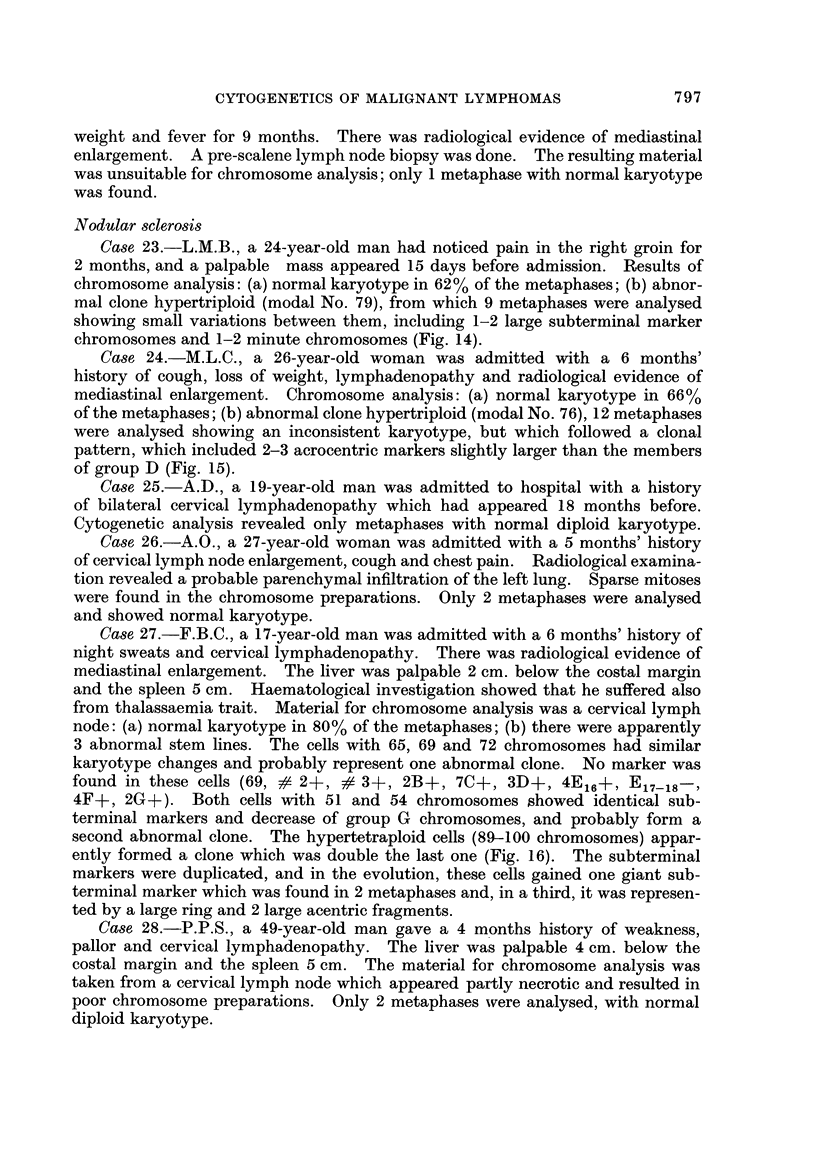

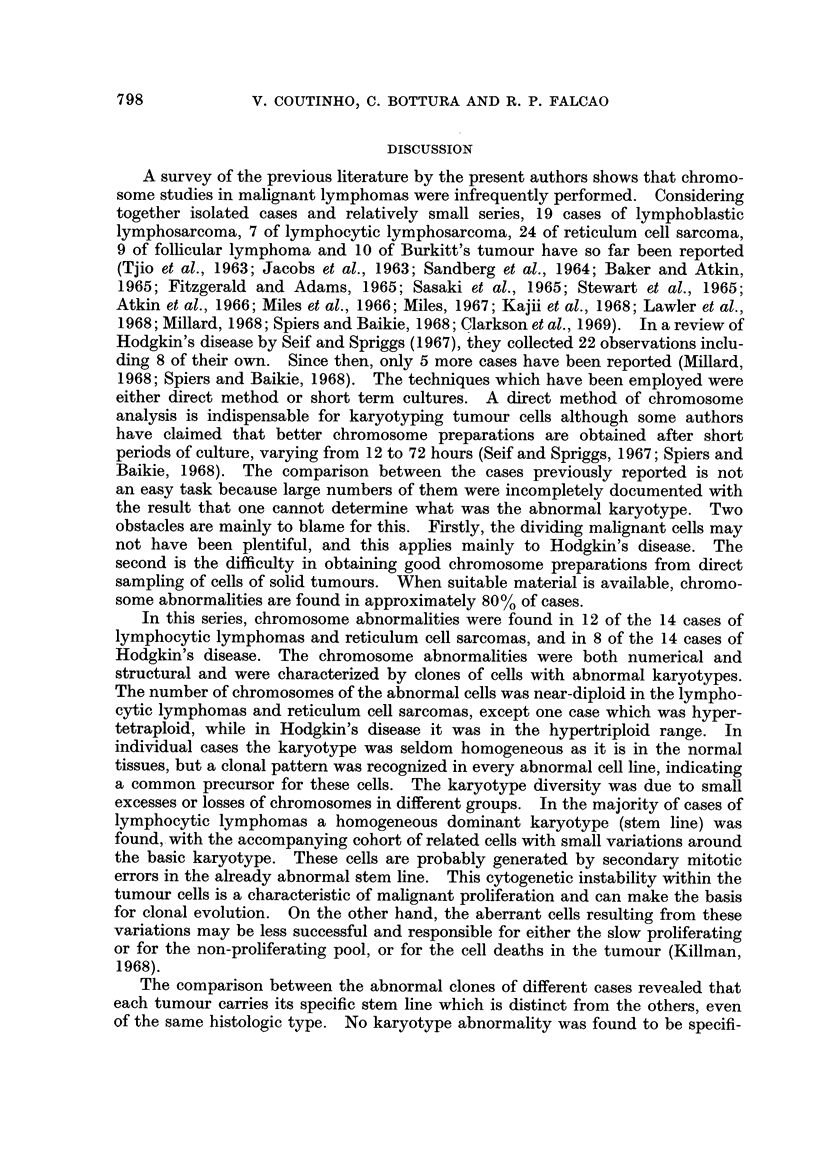

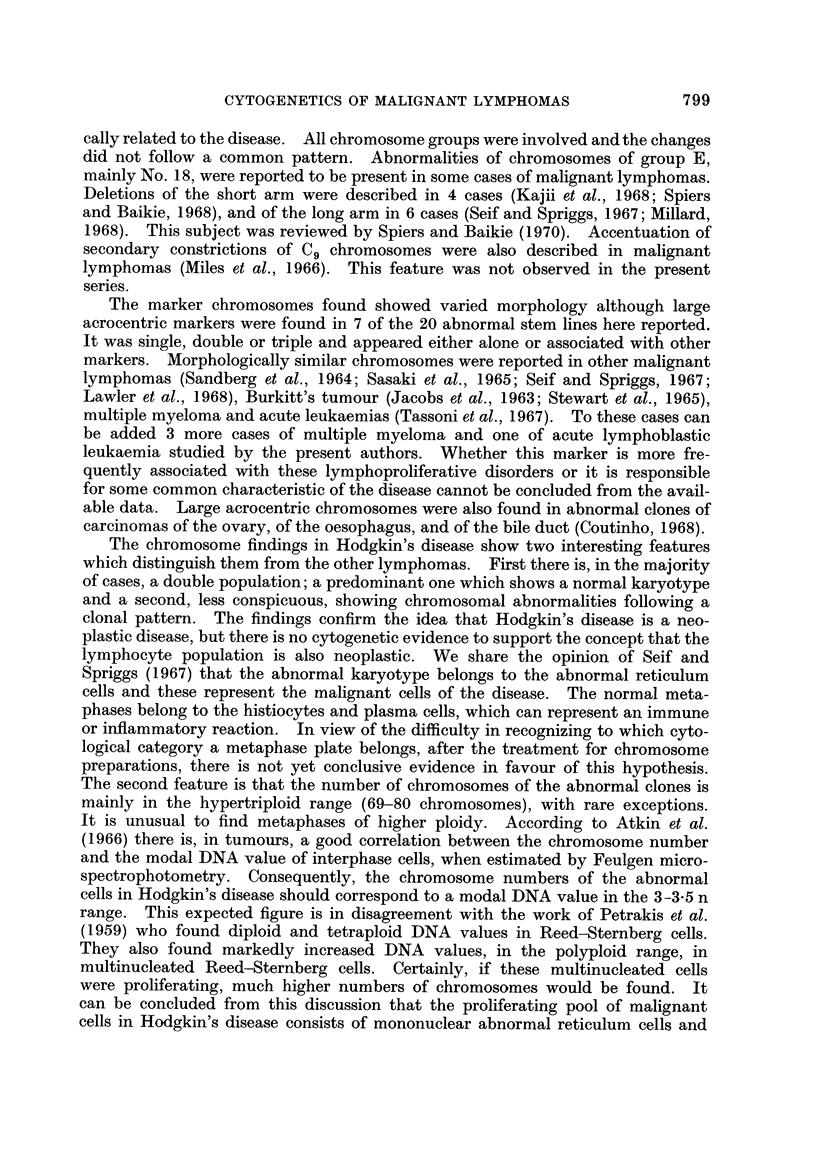

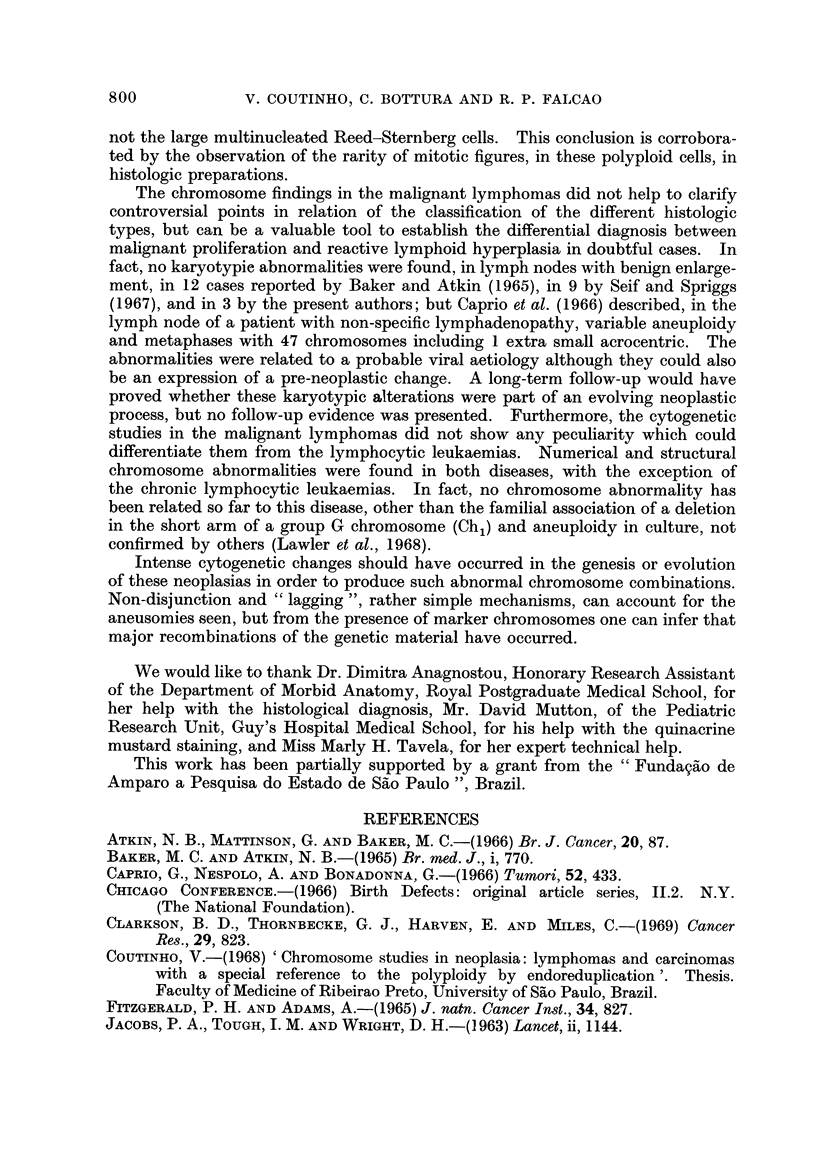

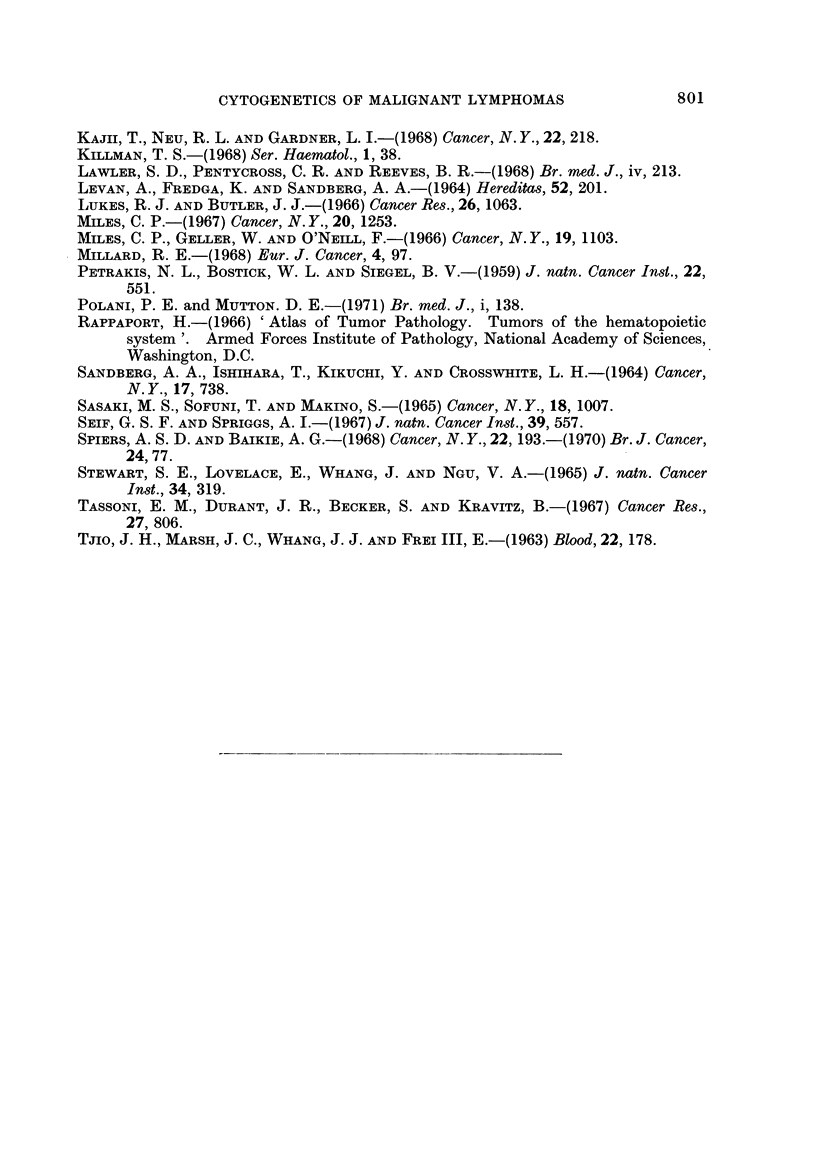


## References

[OCR_01412] BAKER M. C., ATKIN N. B. (1965). CHROMOSOMES IN SHORT-TERM CULTURES OF LYMPHOID TISSUE FROM PATIENTS WITH RETICULOSIS.. Br Med J.

[OCR_01416] Caprio G., Nespolo A., Bonadonna G. (1966). Anomalie cromosomiche in un linfonodo non neoplastico.. Tumori.

[OCR_01420] Clarkson B. D., Thorbecke G. J., de Harven E., Miles C. (1969). Immunoglobulin synthesis by human reticulum sarcoma cells in vivo and during long-term culture in vitro.. Cancer Res.

[OCR_01428] Fitzgerald P. H., Adams A. (1965). Chromosome studies in chronic lymphocytic leukemia and lymphosarcoma.. J Natl Cancer Inst.

[OCR_01434] Kajii T., Neu R. L., Gardner L. I. (1968). Chromosome abnormalities in lymph node cells from patient with familial lymphoma. Loss of No. 3 chromosome and presence of large submetacentric chromosome in reticulum cell sarcoma tissue.. Cancer.

[OCR_01436] Lawler S. D., Pentycross C. R., Reeves B. R. (1968). Chromosomes and transformation of lymphocytes in lymphoproliferative disorders.. Br Med J.

[OCR_01457] SANDBERG A. A., ISHIHARA T., KIKUCHI Y., CROSSWHITE L. H. (1964). CHROMOSOMES OF LYMPHOSARCOMA AND CANCER CELLS IN BONE MARROW.. Cancer.

[OCR_01466] STEWART S. E., LOVELACE E., WHANG J. J., NGU V. A. (1965). BURKITT TUMOR: TISSUE CULTURE, CYTOGENETIC AND VIRUS STUDIES.. J Natl Cancer Inst.

[OCR_01460] Seif G. S., Spriggs A. I. (1967). Chromosome changes in Hodgkin's disease.. J Natl Cancer Inst.

[OCR_01474] TJIO J. H., MARSH J. C., WHANG J., FREI F. (1963). ABNORMAL KARYOTYPE FINDINGS IN BONE MARROW AND LYMPH NODE ASPIRATES OF A PATIENT WITH MALIGNANT LYMPHOMA.. Blood.

[OCR_01470] Tassoni E. M., Durant J. R., Becker S., Kravitz B. (1967). Cytogenetic studies in multiple myeloma: a study of fourteen cases.. Cancer Res.

